# GelInsight: Open-source software for large-sample DNA fragmentation quality control in gel electrophoresis images

**DOI:** 10.1371/journal.pone.0340374

**Published:** 2026-01-07

**Authors:** Kathlyne Jayne B. Bautista, Marjan Mehrab-Mohseni, Sultan A. Kiradoh, Paul A. Dayton, Samantha G. Pattenden

**Affiliations:** 1 Lampe Joint Department of Biomedical Engineering, The University of North Carolina and North Carolina State University, Chapel Hill, North Carolina, United States of America; 2 Division of Chemical Biology and Medicinal Chemistry, Eshelman School of Pharmacy, The University of North Carolina, Chapel Hill, North Carolina, United States of America; 3 Lineberger Comprehensive Cancer Center, The University of North Carolina, Chapel Hill, North Carolina, United States of America; Islamic Azad University, IRAN, ISLAMIC REPUBLIC OF

## Abstract

High throughput DNA fragmentation technology for next generation sequencing have become widely available, but there remains a need for affordable and efficient DNA fragmentation pattern analysis. Commercial electrophoresis platforms, such as the TapeStation, are costly, time-consuming, and have limited batch-processing capabilities. Traditional gel electrophoresis provides a low-cost, high-throughput alternative. However, existing open-source software, such as ImageJ, for gel electrophoresis image analysis typically requires extensive manual pre-processing and yields limited quantitative metrics relevant to DNA fragmentation quality control. Here, we have developed an open-source MATLAB-based software, *GelInsight*, for bulk analysis of gel electrophoresis images for analysis and quality control of DNA fragmentation patterns. *GelInsight* integrates automated image and signal processing tools to determine the base pair size distribution of each sample and to calculate key quality control metrics, including multiple peak base pair sizes and base pair size percentage within a specified range. A user-friendly graphical user interface facilitates efficient data interaction and comprehensive visualization of the analytical outputs. The quantification accuracy of *GelInsight*, including peak base-pair accuracy and relative area measurements, is consistent with both existing open-source software (within 2 ± 2 bp) and commercial assays (within 64 ± 24 bp). Overall, this automated tool streamlines gel image analysis and enhances reproducibility and quantitative rigor in assessing DNA fragmentation patterns.

## Introduction

Next-generation sequencing (NGS) has continued to revolutionize biology and medicine in recent years. NGS uses massively parallel sequencing technology that enables ultra-high throughput sequencing of millions to trillions of DNA fragments within a single instrument run [1]. Sequencing an entire human genome can now be accomplished within a single day [2]. The advancements of NGS in scalability, speed, and accuracy have led to important applications in precision medicine [3] and in the study of novel pathogens [4,5] and diseases [6,7].

Random, unbiased fragmentation or shearing of DNA samples is a crucial first step in the NGS sample preparation pipeline. Downstream analyses require DNA efficiently sheared to consistent fragment sizes determined by the sequencing platform, which can be as small as 100 base pairs (bp) [8]. To avoid sequence bias and ensure accurate representation of the genome in the sequencing data, unbiased fragmentation is necessary [9]. A method combining a benchtop ultrasonic water bath with perfluorocarbon nanodroplets as cavitation enhancing agents demonstrates rapid and consistent DNA fragmentation comparable to sonication using a dedicated commercial sonicator [10]. We have scaled up the application of this method to sonicate DNA samples in a 96-well plate, substantially increasing throughput while maintaining unbiased and consistent fragmentation (see companion manuscript, Mehrab-Mosheni, *et al*., A high throughput system that fragments 96 samples of genomic DNA in parallel for next generation sequencing library preparation).

As high-throughput DNA fragmentation technologies become more widely available, affordable, and efficient systems are needed to determine DNA fragmentation patterns and perform quality control in many samples. The cost of analyzing samples in commercially available systems, such as the TapeStation, quickly increases with higher sample numbers in addition to the initial high purchase price of the system. Analysis using benchtop gel electrophoresis provides an affordable alternative that can accommodate large sample numbers given the prevalence of the technique and the ease of use in laboratories. However, analysis of DNA fragmentation patterns from gel electrophoresis images requires computation using dedicated software. Specifically relevant to NGS applications, fragmented DNA appears on the gel as “smear” with a range of fragment sizes instead of a discrete band. Available open-source software, such as ImageJ [11,12] require manual multi-step pre-processing and time-consuming manipulation of the images to outline each band of interest. In addition, post-processing outside of the software is typically required to extract relevant DNA fragmentation metrics, such as peak base pair size and size distribution. There remains a need for automated batch processing of gel electrophoresis images of fragmented DNA to efficiently extract quantifiable quality control metrics.

Here, we describe *GelInsight*, an open-source software based in MATLAB, for automated and efficient processing of gel electrophoresis images to extract and analyze patterns in fragmented DNA samples. The software implements image processing and signal analysis techniques to automatically determine the base-pair size distribution, peak base-pair size, and other quality control metrics in multiple samples. Minimal user input is required, and a graphical user interface (GUI) allows for easy interaction with the presented data.

We strongly recommend that readers visit the GitHub repository (https://github.com/kjbautista/gelinsight) for the latest version of the software and for detailed documentation.

## Software description

*GelInsight* is available as a standalone app for Windows (under Releases in the GitHub repository). The GitHub repository provides the source code, developed in MATLAB R2020b, which can be implemented on any operating system. Implementation through MATLAB requires the Image Processing and Signal Processing Toolboxes. The software will undergo constant development according to user needs. Copyright is owned by the University of North Carolina at Chapel Hill, and use is free for non-profit research applications or may be licensed for commercial purposes.

The *GelInsight* workflow includes three primary steps. First, the user provides the gel electrophoresis image file and crops the image to a smaller region of interest (ROI). This pre-processing step removes unnecessary information and reduces the image size that will be analyzed by the software for quicker analysis. Second, the user specifies image-specific parameters to ensure accuracy. These parameters include the number of samples in the ROI, the location of the ladder relative to the other samples, the ladder base-pair values, target base-pair range, and the amount of noise reduction to apply (defined as the smoothing factor in the software). Given the number of samples in the ROI and ladder base-pair values, the software automatically detects the lane boundaries and the location of each marker in the ladder. The user can manually adjust the lane boundaries and marker locations in the case of inaccurate detections. The software proceeds to analysis only after the user has confirmed the lane and marker detections. In the last step of the workflow, the software automatically analyzes each sample. Analysis includes quantification of base-pair size distribution of the sample and determination of the peak base-pair size. In the case of multiple base-pair peaks within a single sample, the software calculates the relative base-pair area percentage for each detected peak. As a quality control metric for DNA fragmentation, the software also provides the percentage of the size distribution within a user-defined target base-pair range. Compared with currently available software, *GelInsight* requires minimal user input and automates the use of image processing and signal analysis tools.

*GelInsight* returns the results of the analysis as plots and tables through the interactive GUI. For each sample, the user can visualize the size distribution and the detected peaks. A table summarizes the peak base-pair values with corresponding area percentages. A bar plot displays the percentage of each sample within the target base-pair range. Finally, the user has the option to export the plots as images and the tabular data for further analysis. The raw base-pair size distribution data is also available for secondary analysis by the user.

## Implementation

The following subsections detail the programmatic implementation of the software.

### Image pre-processing

Accurate edge detection and size quantification from gel electrophoresis images in downstream analyses requires reliable image quality. Here, we employ a series of image processing techniques to remove artifacts and enhance the quality of the input image using functions provided by the Image Processing Toolbox in MATLAB.

Sufficient removal of artifacts while retaining the edge features associated with the lane boundaries and the ladder bands is required for consistent lane and ladder detections. We apply a morphological reconstruction algorithm [13] (*imfill*) to fill any holes in the image. To reduce Gaussian noise while preserving the edges, we apply a median filter (*medfilt2*) followed by a pixel-wise adaptive Weiner-based filter (*wiener2*) [14]. The adaptive filter estimates the local variance in the image and applies pixel-wise smoothing based on the estimated local variance.

**Fig 1:**
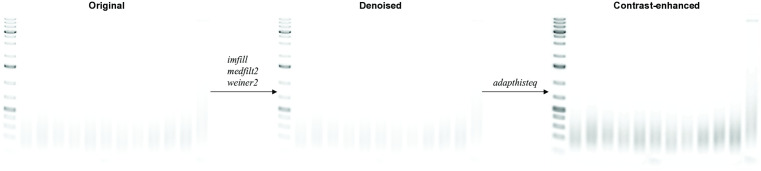
Image processing removes artifacts and improves visualization. A series of image processing algorithms is applied to the original region of interest (left) selected by the user. *imfill* was applied to remove any hole artifacts, followed by *medfilt2* and *weiner2* to remove Gaussian noise adaptively. The result is the denoised image (middle). To improve visualization for the user, *adapthisteq* is applied to adaptively improve the contrast of the ladder markers and sample bands from the background, generating a contrast-enhanced image (right).

To improve visualization, we enhance the contrast using contrast-limited adaptive histogram equalization (*adapthisteq*) [15]. This adaptive algorithm operates on smaller regions to avoid amplification of any noise artifacts while improving contrast regionally to prevent image saturation. The purpose of this contrast-enhancement step is to improve visualization of the individual ladder bands and samples, so the user can better confirm the lane and ladder detections provided by the software. Base-pair size quantification of samples is performed on the denoised image, not on the contrast-enhanced image. Fig 1 summarizes the image pre-processing steps.

## Lane detection

**Fig 2:**
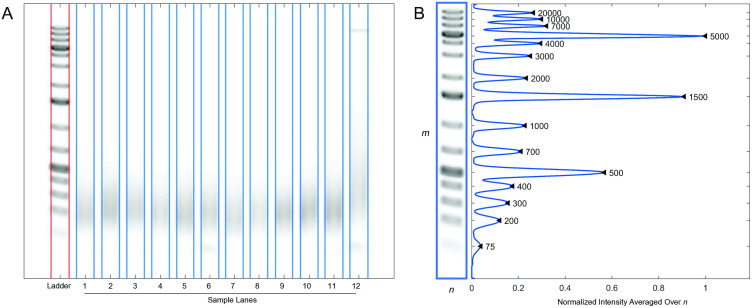
The lane boundaries and the locations of the Thermo Scientific GeneRuler 1 kb Plus DNA ladder markers are automatically detected by the software. (A) All edges in the images are identified using a non-selective Canny edge detector, and the Sobel filter selectively isolates the vertical edges, identifying the lane boundaries for the ladder (*red*) and the sample lanes (*blue*). (B) Given the user-confirmed location of the ladder region *m* ´ *n*, the average intensity of the ladder across the pixel rows *m* is averaged over *n*. Peak detection finds the pixel location of each marker in *m*. The black triangles with corresponding base-pair values shows the peak detections for each ladder marker.

Automatic edge detection to determine the boundaries of each lane is implemented in two steps. First, a Canny-based edge detection is performed on the denoised image. The Canny method finds edges by calculating the local maxima of the gradient of the input image and using a high and a low edge threshold [16]. The two-threshold approach improves the robustness and sensitivity of the edge detection. The thresholds are calculated heuristically. Next, secondary edge detection is applied on the Canny-detected edges to isolate the vertical edges using a gradient-magnitude method using the Sobel approximation [17]. This step assumes that each lane is oriented vertically in the image. Based on the total number of lanes *k* indicated by the user, the software determines the 2 × *k* strongest edges detected by the edge detection algorithm as the left and right boundaries of the lanes (Fig 2A).

### Ladder marker localization

The intensity of the reference ladder lane region with the size, where *m* is the number of pixel rows and *n* is the number of pixel columns, is average across *n*. The normalized result provides the intensity curve of the ladder along *m*. The *findpeaks* function from the Signal Processing Toolbox is used to find the local maxima along the curve, where each local maximum corresponds to the location of a ladder marker (Fig 2B). Peak detection parameters are determined heuristically. Using linear interpolation (*interp1*), the base-pair values of each pixel row in the image are then calculated from the ladder marker localizations and their corresponding user-defined base-pair values.

### Size quantification

Following the same principles implemented in the ladder marker localization, for each sample lane region with the size *x* × *y*, the intensity is first averaged across *y*, generating a sample intensity curve along *x*. Then, the base-pair values of each pixel in *x* are determined from the interpolated base-pair pixel row values. The result is an intensity curve as a function of base-pair sizes.

To reduce noise in the intensity signal, a user-defined smoothing factor is used to apply weighted smoothing is to the intensity curve defined by,


$$I={{s\times I}_{smooth}+(\text{1}-s)\times I}_{raw}$$

where *I* is the final intensity used for analysis, *s* is a smoothing factor value between 0 and 1, *I*_*smooth*_ is the smoothed intensity from a Gaussian-weighted moving average window, and *I*_*raw*_ is the raw intensity curve. Since the amount of noise present in the intensity curve may vary from image to image, the user has the option to set the degree of smoothing from 0 (no smoothing) to 1 (full smoothing) with the smoothing factor.

### Quality control metrics

From the intensity curve, we calculate quality control metrics to evaluate the efficacy of DNA fragmentation in each sample. First, the peak base-pairs are determined using *findpeaks.* When multiple peaks are detected, the relative area under the intensity curve is estimated for each peak using trapezoidal numerical integration (*trapz*). As an additional quality control metric, the percentage of the fragmented sample bands within a target base-pair size range is calculated using trapezoidal numerical integration.

## Demonstration

**Fig 3:**
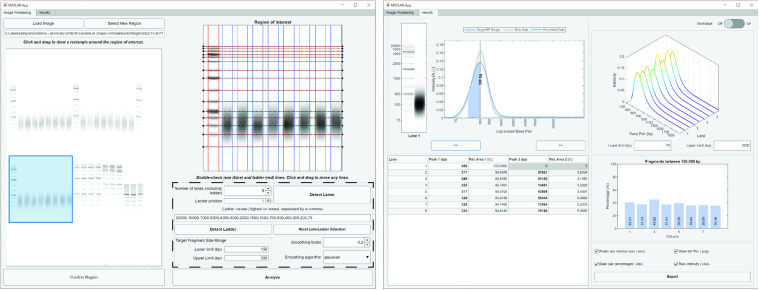
Summary of the *GelInsight* graphical user interface. The Image Processing tab (*left*) displays the input image, and the region of interest annotated with the lane boundary and ladder marker detections. The user input panel is outlined with a black dashed line. Following analysis, the software switches to the Results tab (*right*) where a table and several plots display the DNA fragmentation pattern analysis result for each sample in the region of interest.

The *GelInsight* GUI provides a guided, straightforward design that facilitates use and interpretability of the software. The GUI guides the user through the initial user input stage through the analysis, and informative plots and tables then detail the results following analysis. Fig 3 provides representative screenshots of the software in use.

### Experimental validation

To benchmark the accuracy of *GelInsight* peak base-pair determinations, we analyzed three different DNA ladders as a function of agarose percentage in the gel: New England Biolabs (NEB) 1kb (500 to 10000 bp), Invitrogen 100 bp (100–2000 bp), and Invitrogen 1 kb plus (100 to 15000 bp). These ladders contain chromatography-purified DNA fragments and generate high-contrast and high-quality bands in the gel with known sizes. The Invitrogen 1 kb plus ladder was used as the reference. We quantified percent error for each sample band in each ladder, defined by,


$$\text{Percent}\text{error}=\frac{{|\text{peak}}_{\text{measured}}-{\text{peak}}_{\text{reference}}|}{{\text{peak}}_{\text{reference}}}\times \text{1}\text{0}\text{0}\%.$$

**Fig 4:**
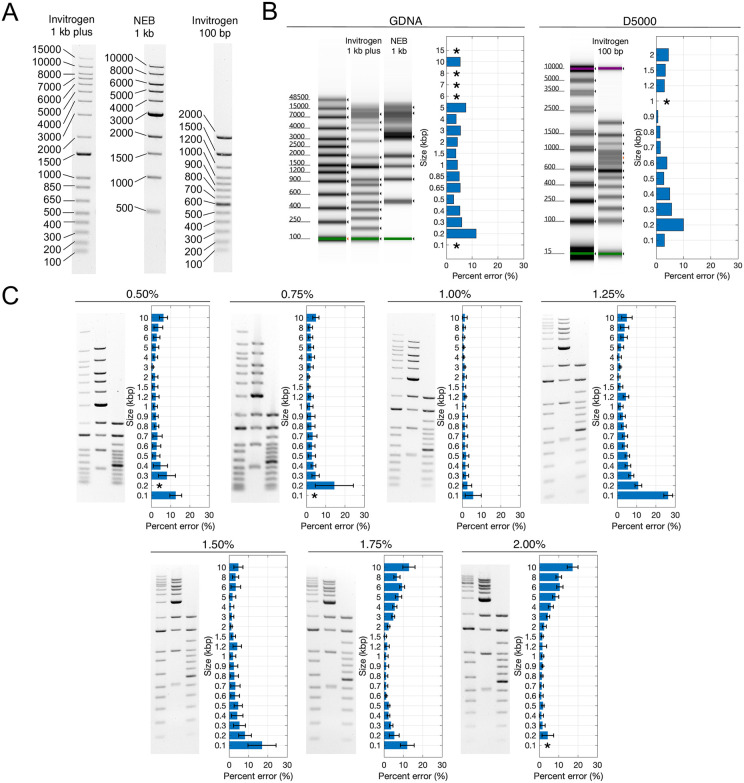
Peak base pair measurement accuracy using *GelInsight* as a function of agarose percentage. (A) Representative ladders used in the analysis: Invitrogen 1 kb plus, Invitrogen 100 bp, New England Biolabs (NEB) 1 kb. (B) TapeStation results and peak detection errors for each band in the DNA ladders, measured from the Genomic DNA (GDNA) and the D5000 ScreenTape Assays. (C) Average *GelInsight* peak detection errors for each band in the sample DNA ladders across *N* = 3 replicates for a range of agarose percentages, from 0.50% to 2.00%. Invitrogen 1 kb plus was used as the reference ladder, while Invitrogen 100 bp and NEB 1 kb were used as the sample ladders. The asterisk (*) denotes missed peaks detections.

The accuracy of the peak base-pair determinations provided by *GelInsight* for samples ranging from 100 to 10000 bp are summarized in Fig 4. The ladders included in the analysis and the nominal size of each band are provided in Fig 4A. To compare against a standard method, the size of each sample in the ladder was measured using an Agilent Genomic DNA (GDNA) or D5000 Screen TapeStation system (Agilent Technologies, CA, USA) (S2 File). For *GelInsight* quantification, the ladders were loaded and ran on gels of different agarose percentages (from 0.50% to 2.00%) for *N* = 3 replicates. The average percent error of the size determined by the TapeStation assays and *GelInsight* relative to the nominal size for each band in the sample ladders are calculated in Fig 4B and Fig 4C, respectively. For target sizes that are duplicated across ladders (*e.g.*, Invitrogen 100 bp and NEB 1 kb both contain a 500-bp band), the average peak measurement is used.

The accuracy of detecting these calibrated DNA fragments using *GelInsight* depends on the agarose percentage. For agarose percentages less than 1%, bands less than 1000 bp have decreased spatial separation, resulting in lower peak accuracy and missed peak detections. Conversely, agarose percentages greater than 1.50% resulted in low peak accuracy for the bands greater than 2000 bp. The same trends are observed in the TapeStation assays. Poor separation of bands greater than 2500 bp in the GDNA assay and bands less than 1000 bp in the D5000 assay resulted in a large number of missed peaks. The contrast of the band relative to the gel background also affects *GelInsight*’s peak accuracy. In the 1.25% and 1.50% agarose gels, bands less than 500 bp appear more diffused and have low contrast. The resulting blurred intensity curve and poor contrast likely reduced the accuracy of peak detection even though the bands have high electrophoretic separation. For 1% agarose percentage, *GelInsight* most accurately measured the peak base-pair for most of the ladder bands with average percent errors less than 5 to 10%. These results suggest that the peak detection accuracy of the software strongly depends on the quality of gel electrophoresis experiment result, similar to using the TapeStation where quantification accuracy relies on selecting an assay with the appropriate linear range.

To validate the experimental utility of *GelInsight*, we analyzed *N* = 3 replicates of gel electrophoresis images of fragmented DNA samples (*n* = 8) on the software. Ten nanograms per microliter (ng/µL) of human genomic DNA (gDNA) was sonicated in Covaris microtubes with a final volume of 100 μl TE. Covaris LE220 was operated with a peak indicate power of 450 W, duty factor of 20%, and cycle per burst of 200. DNA fragment quality and size were assessed using an Agilent D5000 or D1000 Screen TapeStation assays and using 1.5% agarose gel analyzed with *GelInsight* and ImageJ. *GelInsight* was used to calculate the base-pair peak and relative peak area under the curve. The TapeStation interface similarly provides the base-pair peaks and integrated area. ImageJ, a commonly used open-source software for manual gel electrophoresis image analysis, was also used for peak base-pair and area under the curve analysis [11]. Using the *Plot Lanes* function in the built-in *Gel Analyzer* plugin [12], ROIs were manually drawn to outline each individual lane. Implementing the procedure reported by Craig et al. [18], intensity profiles were extracted for each reference ladder and sample. The peak of each sample profile was approximated by identifying the location of maximum intensity and applying piece-wise interpolation in MATLAB to calculate the corresponding base-pair size relative to the intensity profile and peaks of the reference ladder. The built-in *Analyze* function was used to measure the relative area under the curve of each peak by manually defining the bounds of the peak along the intensity profile, calculating the area under the curve within these bounds, and normalizing this value by the area under the curve of the entire intensity profile.

**Fig 5:**
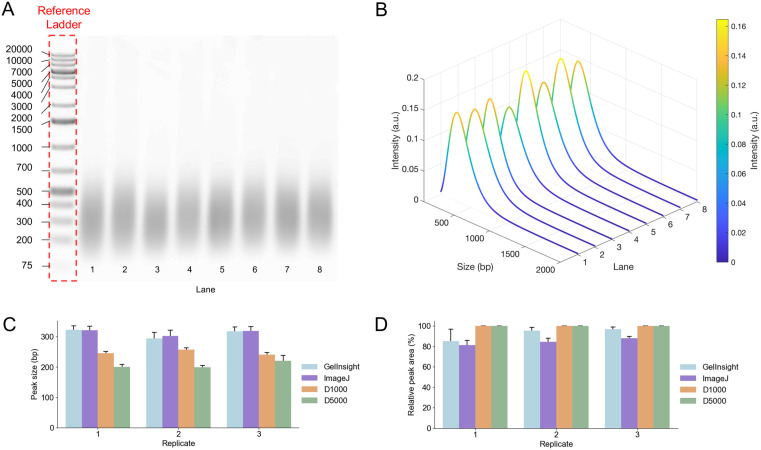
Comparison of peak base pair detection using *GelInsight* with standard methods. (A) Representative gel electrophoresis image of sonicated DNA samples, and (B) corresponding waterfall plots displaying the base-pair intensity curves for each DNA sample. (C) The average peak base pair across the DNA samples (*n* = 8) using *GelInsight*, ImageJ analysis, and TapeStation ScreenTape assays (D1000 and D5000) for each replicate (*N* = 3). (D) The relative peak area measurements across the DNA samples (*n* = 8) using *GelInsight*, ImageJ analysis, and TapeStation ScreenTape assays (D1000 and D5000) for each replicate (*N* = 3).

The size quantification and relative peak area results for the sonicated DNA samples are summarized in Fig 5. Across *N* = 3 replicates, we calculated an average base-pair peak of 311 ± 16 bp and 314 ± 16 bp using *GelInsight* and ImageJ, respectively*,* and, in comparison, we calculated average base-pair peaks of 248 ± 7 bp and 207 ± 10 bp using D1000 and D5000 ScreenTape assays on the TapeStation, respectively, (Fig 5C). Like the TapeStation and ImageJ measurements, the base-pair peaks measured by *GelInsight* demonstrate minimal variability less than 20 bp across samples and replicates. *GelInsight* measurements of base-pair peaks are highly consistent with those measured using ImageJ; the average values are within 2 ± 2 bp. Relative to the peak measurements provided by the TapeStation assays, the average percent error of the *GelInsight* peak measurements were 25 ± 10% (64 ± 24 bp) and 51 ± 9% (105 ± 15 bp) for the D1000 and D5000 assays, respectively. Manufacturer specifications define the typical resolution range of the D5000 at 15% between 400 and 15000 bp with a sizing accuracy of ±10%. The average size of the sonicated samples is therefore outside the resolution range of the assay and likely contributed to the significant size difference between the D5000 and the other quantification methods. The D5000 also failed to detect a peak for one of the samples (S3 File). For the D1000 assay, manufacturer specifications define a typical resolution range of 15% between 35 and 300 bp and 10% between 300 and 1000 bp with a sizing accuracy ±10%. The systemic bias between the software-based measurements and the D1000 assay falls within the resolution and accuracy variability range inherent to this TapeStation assay. The variable performance of TapeStation assays can be further observed in the TapeStation analysis of DNA fragments of known size in Fig 4B; the GDNA and D5000 assays reach errors up to 11.5% and 10% respectively. TapeStation accuracy can also decrease with low signal-to-noise profiles as in fragmented DNA smears [19].

The relative peak area results in Fig 5D similarly showed differences between the TapeStation assays and software-based analyses with *GelInsight* and ImageJ. While the TapeStation results consistently yielded 100% relative area under the curve for each peak detected, *GelInsight* and ImageJ provided lower average measurements of 93 ± 6% and 85 ± 3%, respectively. Increased background signal in the gel image can alter the baseline intensity for *GelInsight* and ImageJ analysis, resulting in relative peak area results less than 100%. The relative peak area measurements were further decreased by variabilities introduced by manually drawn ROIs in the ImageJ analysis.

**Fig 6:**
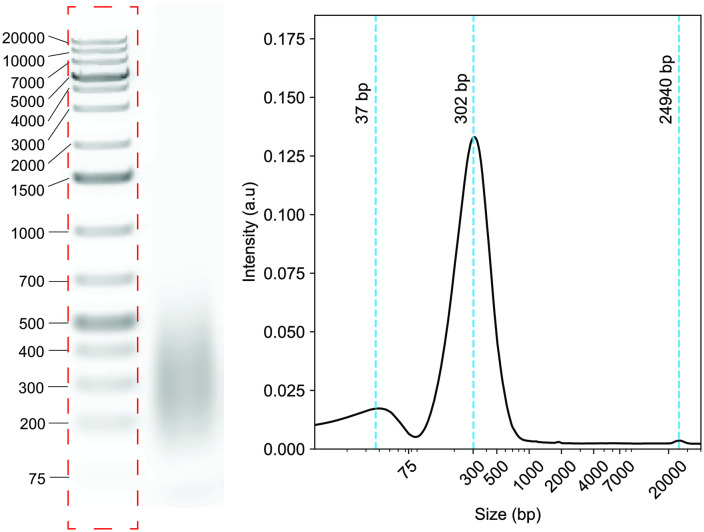
Noisy peaks are detected by *GelInsight.* Example gel electrophoresis image of a sonicated DNA sample with the reference ladder (*left*) and corresponding peak detections by *GelInsight* (*right*).

*GelInsight* may also be more sensitive to low intensity peaks (Fig 6). More peaks are detected by *GelInsight* than by the TapeStation in certain samples. However, in the case of artificial peaks due to artifacts and noise, *GelInsight* may be more likely to label a noise peak as a real peak in the sample. Increasing the smoothing factor parameter can reduce these noisy peak detections. Setting a threshold on the relative peak area percentage, such that peaks below the threshold are attributed to noise, can also separate noise peaks from real peaks in secondary analysis of the software results.

**Fig 7:**
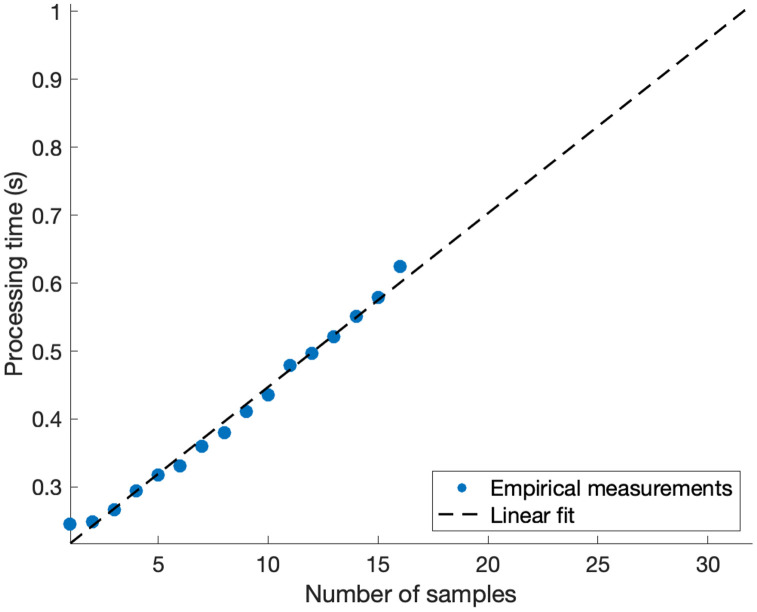
Processing time increases with the number of samples. Processing time as a function of the number of samples, extrapolated up to 32 samples.

To further highlight the use of *GelInsight* as a quality control platform for DNA fragmentation samples, the software calculates an additional metric that determines the percentage of the fragmented sample within a user-defined size range. The average percentage of the fragmented samples measuring between 150 and 600 bp was 92.5% for the sonicated DNA samples, demonstrating highly consistent fragmentation as expected using this sonication technique. There is no hard limit to the number of DNA fragmentation samples that can be analyzed by *GelInsight,* given sufficient computer memory. As a reference, processing 8 samples and 1 reference ladder used approximately 3 GB of computer memory on a standard laptop computer. The processing time also linearly increases with the number of lanes (Fig 7). Each additional sample minimally increases the processing time by 0.03 s, empirically measured on a standard laptop computer. However, this processing time does not include the time required to perform the gel electrophoresis experiment as well as the time required for loading the image and defining the parameters within the software. This initial software setup may add additional time on the order of seconds or minutes depending on user experience.

## Conclusion

As large-sample DNA fragmentation techniques become more commonplace, *GelInsight* insight addresses the need for an accessible and affordable platform for quality control analysis of fragmented DNA samples using gel electrophoresis. *GelInsight* provides an intuitive GUI that requires no prior user training and returns useful quality control metrics, including the base-pair peaks, relative peak area, and the percentage of the fragmented sample within a target size range. The accuracy of *GelInsight* calculations are consistent with expensive commercial platforms, within approximately 60 bp and within the expected variability range of the commercial assays. *GelInsight* performance is also consistent with other gel image analysis tools, such as ImageJ, but with significantly increased efficiency and lower processing time. As an open-source platform, we welcome any modifications and additions requested by the scientific community as we continue to optimize and develop the software.

## Supporting information

S1 File Raw, uncropped gel images from Figs 1–6. (PDF)

S2 File Summary of the TapeStation analysis of the DNA ladders in Fig 4. (PDF)

S3 File Summary of the TapeStation analysis of the sonicated DNA in Figs 5 and 6. (PDF)
