# Supplementary material for: GelInsight: Open-source software for large-sample DNA fragmentation quality control in gel electrophoresis images
Source: PLoS One. 2026 Jan 7;21(1):e0340374. doi: 10.1371/journal.pone.0340374 (PMC12779122; doi:10.1371/journal.pone.0340374)
Supplement: S1 File — (PDF) [file pone.0340374.s001.pdf]

used in Figures 1 and 2; scanned on the Typhoon
1.5% agarose gel

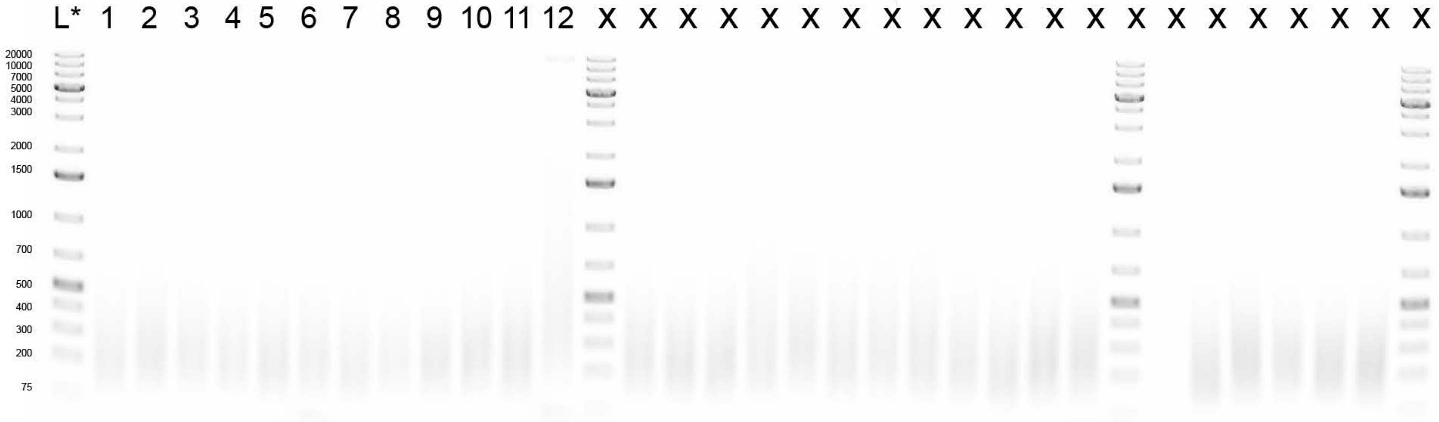


*L = GeneRuler 1 kb plus ladder: 20000,10000,7000,**5000**,4000,3000,2000,**1500**,1000,700,**500**,400,300,200,75 bp
1-12 = fragmented DNA

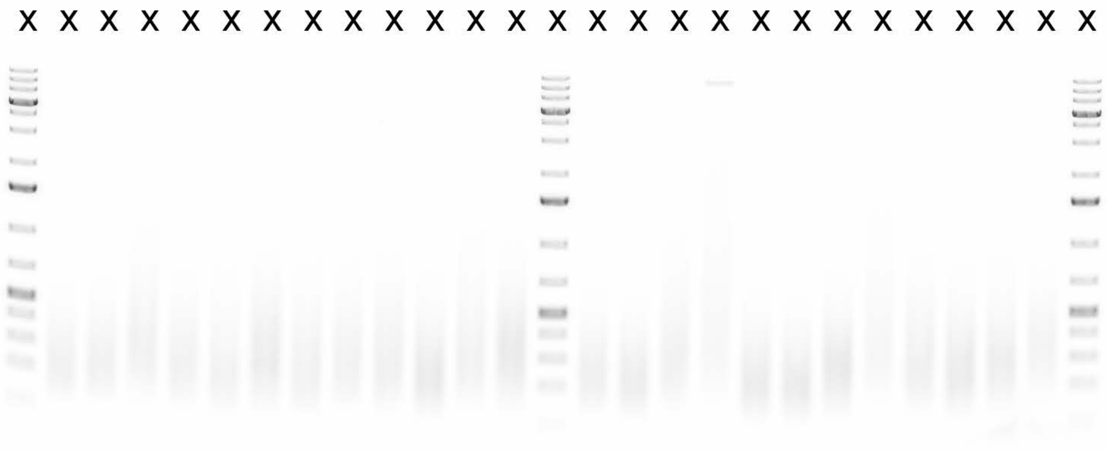


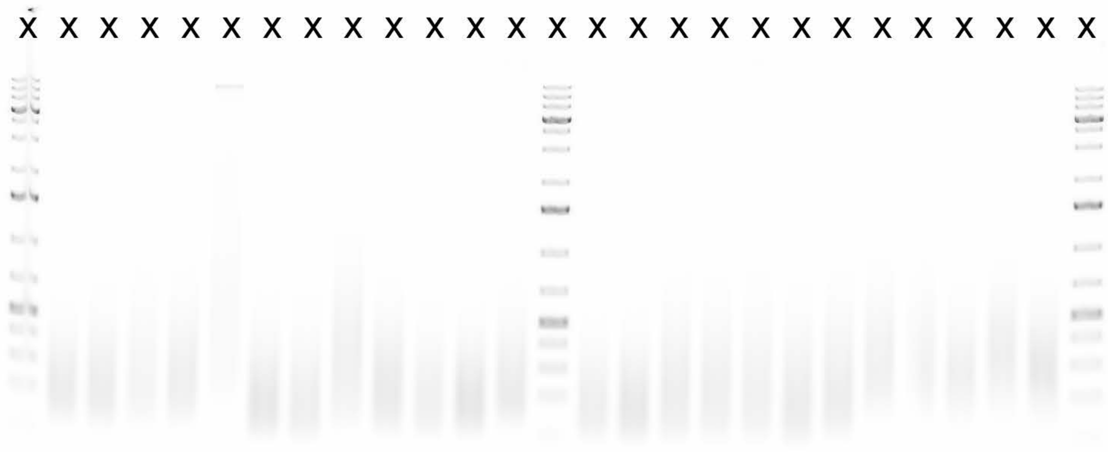

used in Figures 3, 5, and 6; scanned on the Typhoon
1.5% agarose gel

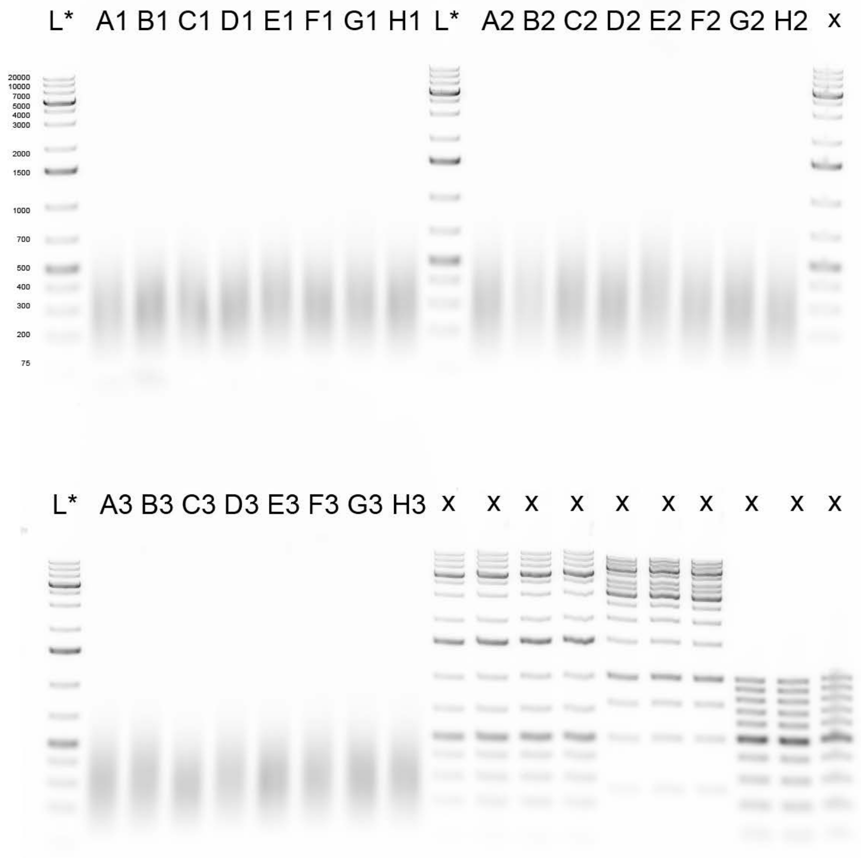


*L = GeneRuler 1 kb plus ladder: 20000,10000,7000,**5000**,4000,3000,2000,**1500**, 1000,700,**500**,400,300,200,75 bp

A1-H1 = sonicated DNA, replicate 1
A2-H2 = sonicated DNA, replicate 2
A3-H3 = sonicated DNA, replicate 3

used in Figure 4; scanned on the UVP Solo Elite using the VisionWorks software package

0.5% agarose gel

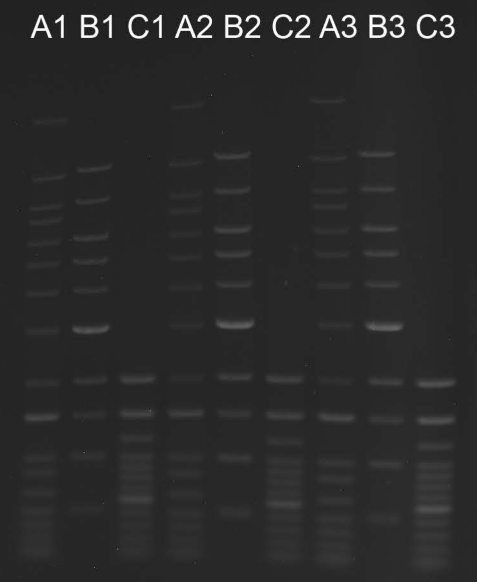


A = Invitrogen 1kb plus ladder: 15000,10000,8000,7000,6000,5000,4000,3000,2000,**1500**,1000,850,650,500,400,300,200,100 bp
B = New England Biolabs 1 kb DNA ladder: 10000,8000,6000,5000,4000,**3000**,2000,1500,1000,500 bp
C = Invitrogen 100 bp: **2000**,**1500**,1200,1000,900,800,700,**600**,500,400,300,200,100 bp

used in Figure 4; scanned on the UVP Solo Elite using the VisionWorks software package

0.75% agarose gel

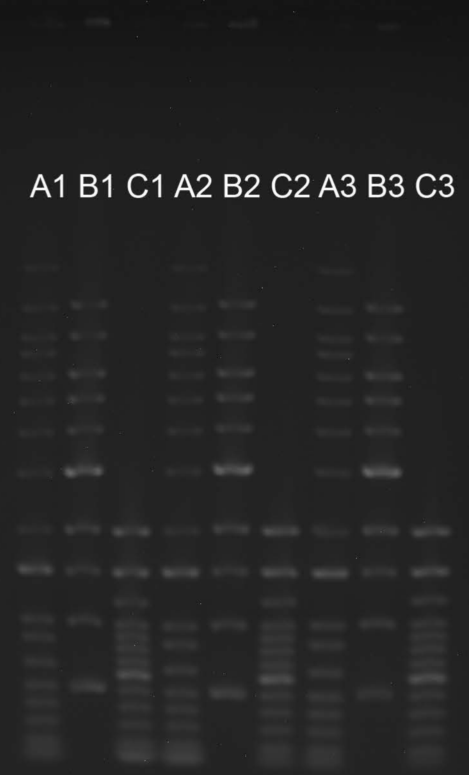


A = Invitrogen 1kb plus ladder: 15000,10000,8000,7000,6000,5000,4000,3000,2000,**1500**,1000,850,650,500,400,300,200,100 bp
B = New England Biolabs 1 kb DNA ladder: 10000,8000,6000,5000,4000,**3000**,2000,1500,1000,500 bp
C = Invitrogen 100 bp: **2000**,**1500**,1200,1000,900,800,700,**600**,500,400,300,200,100 bp

used in Figure 4; scanned on the UVP Solo Elite using the VisionWorks software package

1% agarose gel

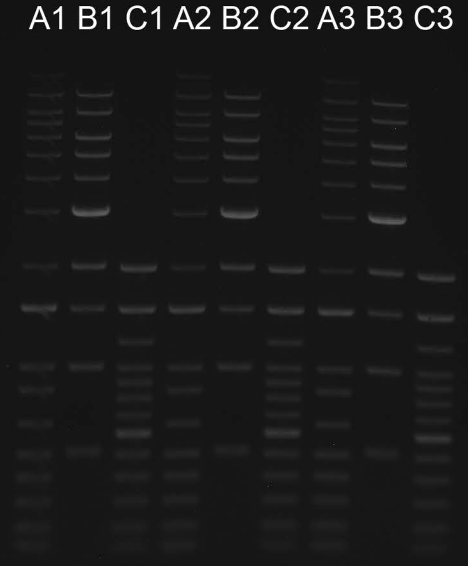


A =Invitrogen 1kb plus ladder: 15000,10000,8000,7000,6000,5000,4000,3000,2000,**1500**,1000,850,650,500,400,300,200,100 bp
B = New England Biolabs 1 kb DNA ladder: 10000,8000,6000,5000,4000,**3000**,2000,1500,1000,500 bp
C = Invitrogen 100 bp: **2000**,**1500**,1200,1000,900,800,700,**600**,500,400,300,200,100 bp

used in Figure 4; scanned on the UVP Solo Elite using the VisionWorks software package

1.25% agarose gel

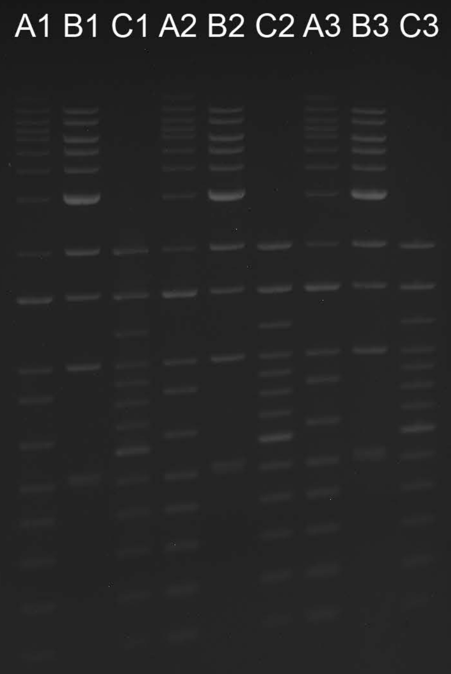


A =Invitrogen 1kb plus ladder: 15000,10000,8000,7000,6000,5000,4000,3000,2000,**1500**,1000,850,650,500,400,300,200,100 bp
B = New England Biolabs 1 kb DNA ladder: 10000,8000,6000,5000,4000,**3000**,2000,1500,1000,500 bp
C = Invitrogen 100 bp: **2000**,**1500**,1200,1000,900,800,700,**600**,500,400,300,200,100 bp

used in Figure 4; scanned on the UVP Solo Elite using the VisionWorks software package

1.5% agarose gel

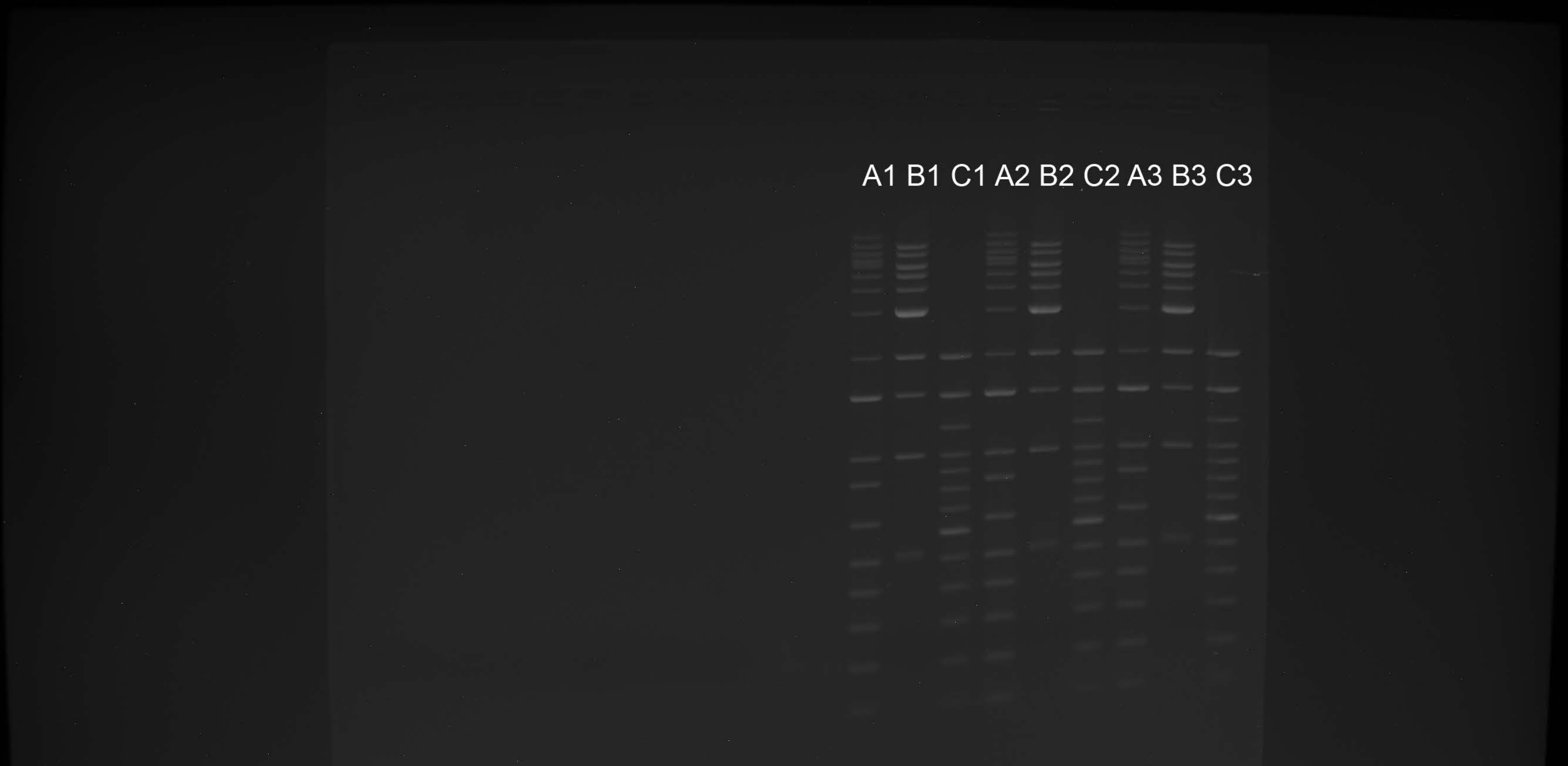


A = Invitrogen 1kb plus ladder: 15000,10000,8000,7000,6000,5000,4000,3000,2000,**1500**,1000,850,650,500,400,300,200,100 bp
B = New England Biolabs 1 kb DNA ladder: 10000,8000,6000,5000,4000,**3000**,2000,1500,1000,500 bp
C = Invitrogen 100 bp: **2000**,**1500**,1200,1000,900,800,700,**600**,500,400,300,200,100 bp

used in Figure 4; scanned on the UVP Solo Elite using the VisionWorks software package

1.75% agarose gel

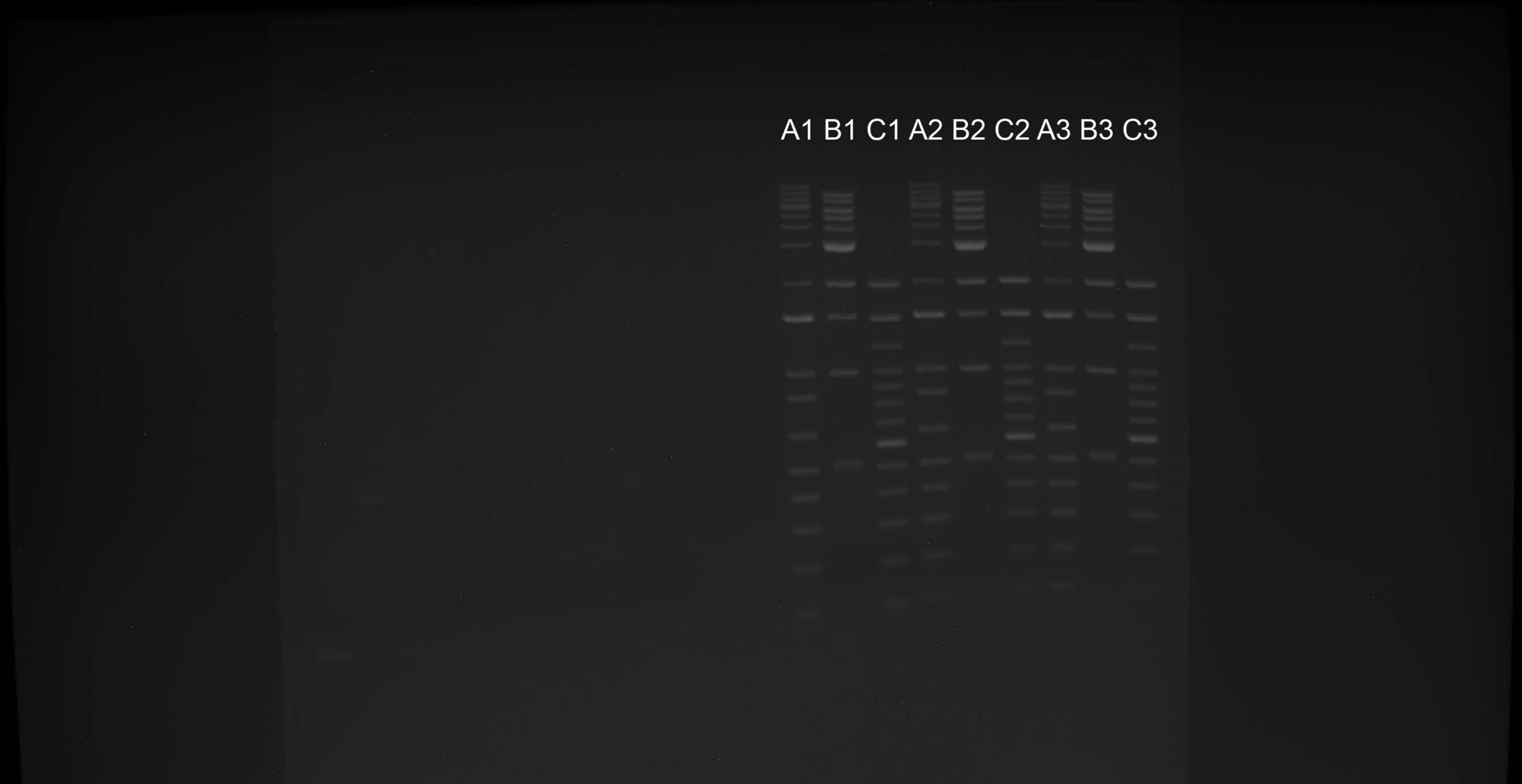


A = Invitrogen 1kb plus ladder: 15000,10000,8000,7000,6000,5000,4000,3000,2000,**1500**,1000,850,650,500,400,300,200,100 bp
B = New England Biolabs 1 kb DNA ladder: 10000,8000,6000,5000,4000,**3000**,2000,1500,1000,500 bp
C = Invitrogen 100 bp: **2000**,**1500**,1200,1000,900,800,700,**600**,500,400,300,200,100 bp

used in Figure 4; scanned on the UVP Solo Elite using the VisionWorks software package

2% agarose gel

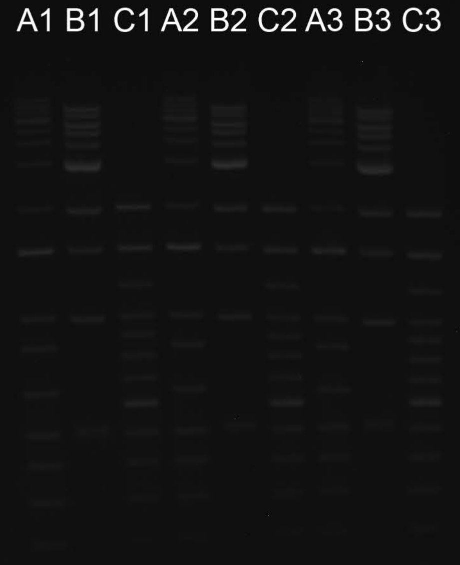


A = Invitrogen 1kb plus ladder: 15000,10000,8000,7000,6000,5000,4000,3000,2000,**1500**,1000,850,650,500,400,300,200,100 bp
B = New England Biolabs 1 kb DNA ladder: 10000,8000,6000,5000,4000,**3000**,2000,1500,1000,500 bp
C = Invitrogen 100 bp: **2000**,**1500**,1200,1000,900,800,700,**600**,500,400,300,200,100 bp
