# Supplementary material for: GelInsight: Open-source software for large-sample DNA fragmentation quality control in gel electrophoresis images
Source: PLoS One. 2026 Jan 7;21(1):e0340374. doi: 10.1371/journal.pone.0340374 (PMC12779122; doi:10.1371/journal.pone.0340374)
Supplement: S2 File — (PDF) [file pone.0340374.s002.pdf]

TapeStation ladder analysis

Genomic DNA ScreenTape assay results

(A) Genomic DNA ScreenTape: sample Information

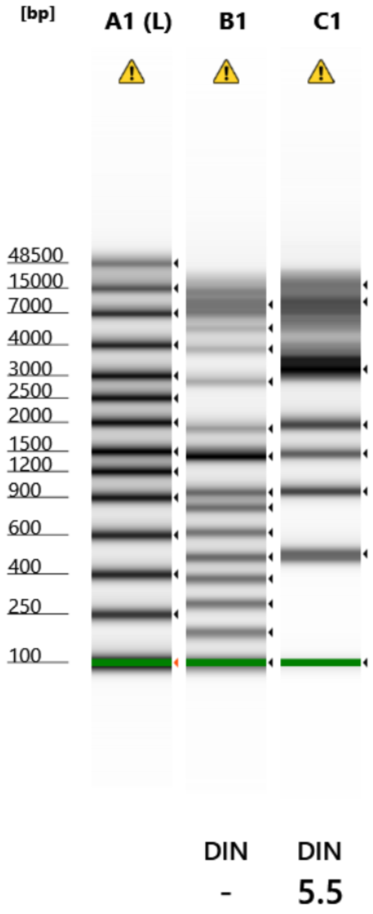

Default image (Contrast 50%), Image is Scaled to Sample

Sample Info

Well	DIN	Conc. [ng/µl]	Sample Description
A1	-	74.6	Ladder
B1	-	91.0	1KB+
C1	5.5	496	1KB

(B) Genomic DNA ScreenTape ladder

A1: Ladder

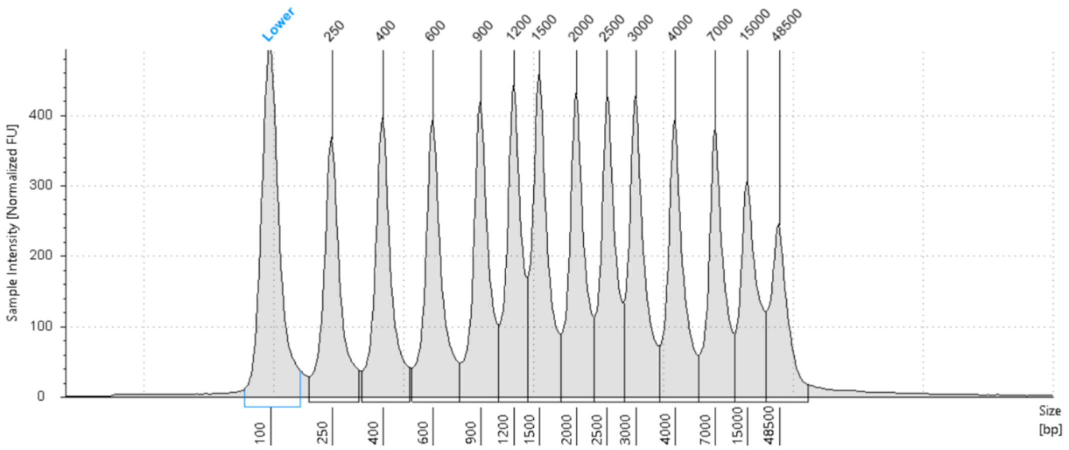

Peak Table

Size [bp]	Calibrated Conc. [ng/µl]	Assigned Conc. [ng/µl]	% Integrated Area	From [bp]	To [bp]	Peak Comment	Observations
100	8.50	8.50	-	67	156		Lower Marker
250	5.62	-	7.55	177	319		
400	5.94	-	7.97	329	496		
600	6.03	-	8.09	505	752		
900	6.13	-	8.22	752	1054		
1200	6.04	-	8.11	1054	1354		
1500	6.66	-	8.94	1354	1768		
2000	6.21	-	8.33	1768	2279		
2500	5.91	-	7.93	2279	2785		
3000	6.13	-	8.23	2785	3560		
4000	5.68	-	7.62	3560	5531		
7000	5.38	-	7.21	5531	11058		
15000	4.85	-	6.50	11058	23721		
48500	3.94	-	5.29	23721	78650		

(C) Invitrogen 1 kb plus ladder

B1: 1KB+

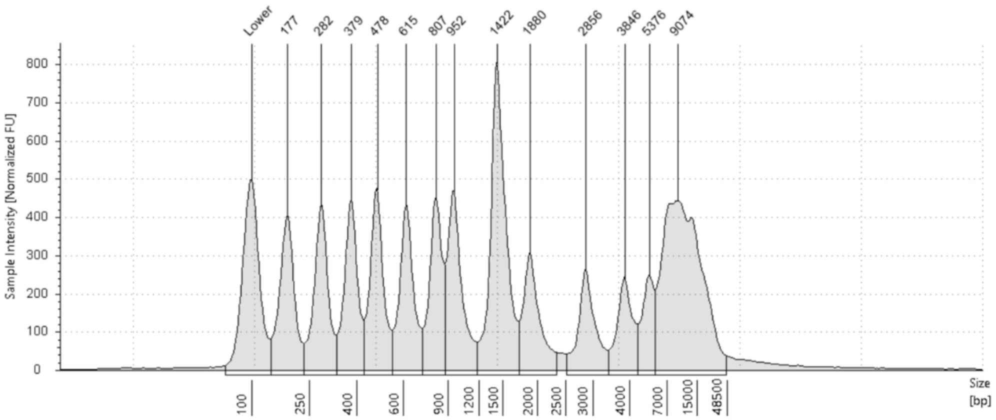

Peak Table

Size [bp]	Calibrated Conc. [ng/µl]	Assigned Conc. [ng/µl]	% Integrated Area	From [bp]	To [bp]	Peak Comment	Observations
100	8.50	8.50	-	66	135		Lower Marker
177	6.09	-	6.74	135	228		
282	6.15	-	6.81	228	327		
379	6.09	-	6.75	327	427		
478	6.43	-	7.12	427	544		
615	6.13	-	6.79	544	711		
807	5.96	-	6.60	711	876		
952	6.98	-	7.73	876	1180		
1422	12.3	-	13.66	1180	1716		
1880	5.01	-	5.54	1716	2325		
2856	4.27	-	4.73	2511	3394		
3846	3.66	-	4.05	3394	4501		
5376	3.14	-	3.48	4501	5875		
9074	18.1	-	20.01	5875	44782		

(D) New England Biolabs 1 kb ladder

C1: 1KB

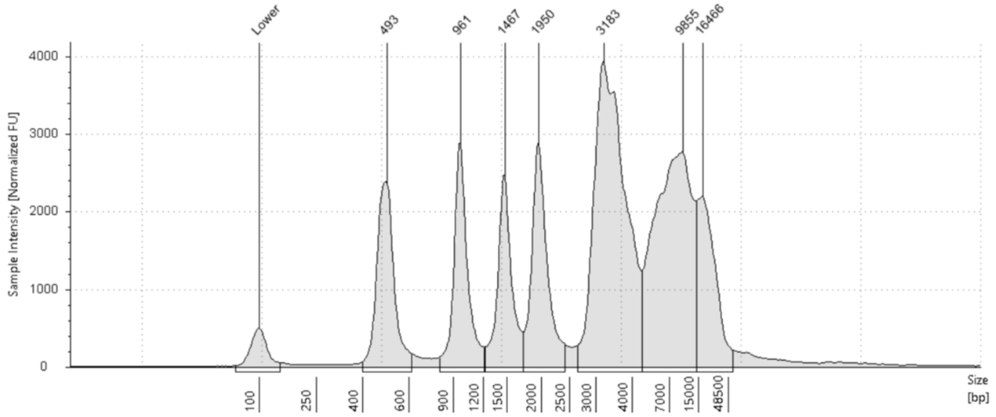

Peak Table

Size [bp]	Calibrated Conc. [ng/µl]	Assigned Conc. [ng/µl]	% Integrated Area	From [bp]	To [bp]	Peak Comment	Observations
100	8.50	8.50	-	68	140		Lower Marker
493	45.7	-	9.45	396	614		
961	42.6	-	8.80	793	1204		
1467	38.8	-	8.02	1215	1722		
1950	50.0	-	10.33	1722	2410		
3183	141	-	29.06	2645	4637		
9855	120	-	24.76	4637	14116		
16466	46.3	-	9.57	14116	54078		

D5000 ScreenTape assay results

(E) D5000 ScreenTape: sample Information

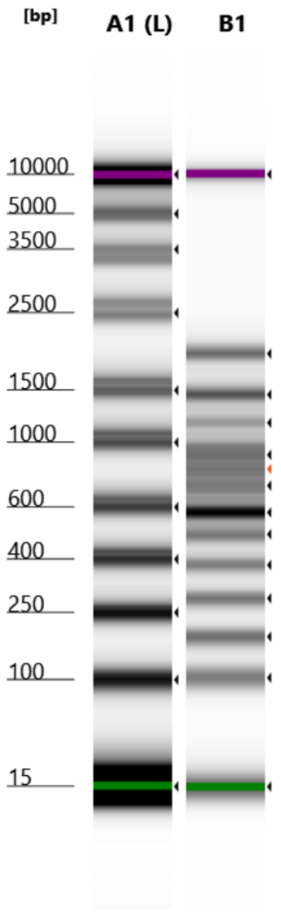

Default image (Contrast 50%), Image is Scaled to Sample

Sample Info

Well	Conc. [ng/µl]	Sample Description
A1	13.1	Ladder
B1	66.5	100bp

(F) D5000 ScreenTape ladder

A1: Ladder

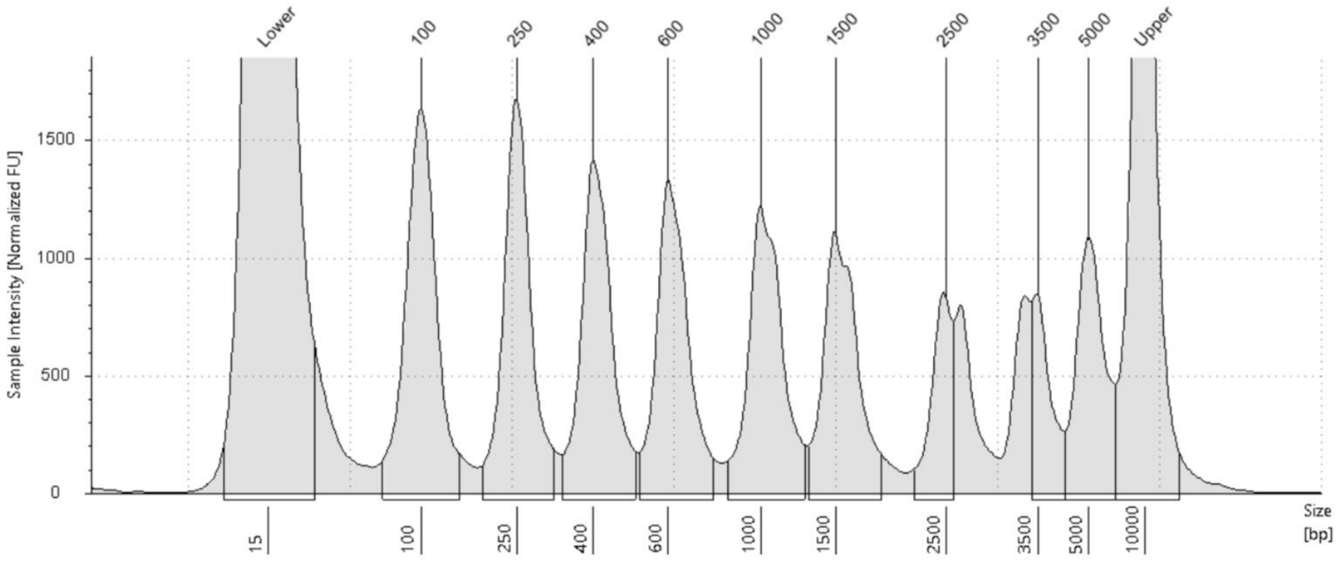

Peak Table

Size [bp]	Calibrated Conc. [ng/µl]	Assigned Conc. [ng/µl]	Peak Molarity [nmol/l]	% Integrated Area	Peak Comment	Observations
15	9.55	-	979	-		Lower Marker
100	2.03	-	31.2	15.46		
250	1.83	-	11.3	13.97		
400	1.74	-	6.71	13.29		
600	1.70	-	4.35	12.92		
1000	1.67	-	2.56	12.69		
1500	1.53	-	1.57	11.67		
2500	0.709	-	0.436	5.40		
3500	0.659	-	0.290	5.02		
5000	1.25	-	0.386	9.56		
10000	3.25	3.25	0.500	-		Upper Marker

(G) Invitrogen 100 bp ladder

B1: 100bp

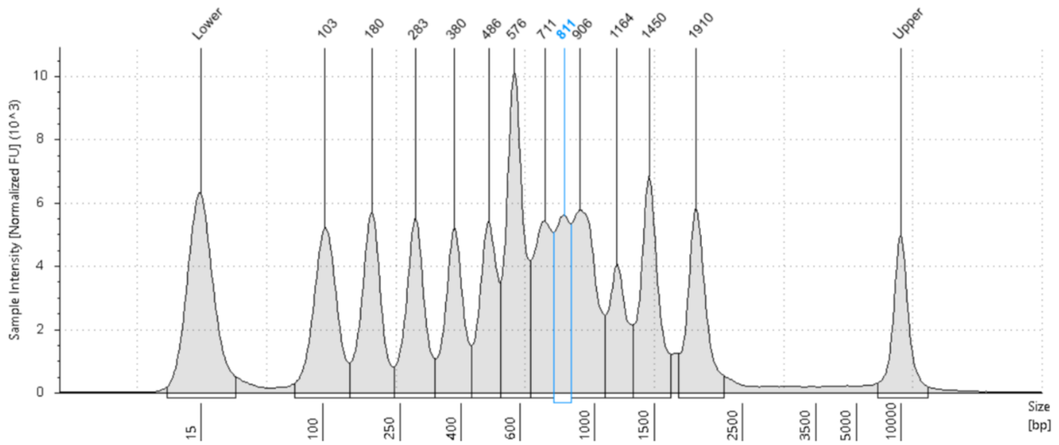

Peak Table

Size [bp]	Calibrated Conc. [ng/µl]	Assigned Conc. [ng/µl]	Peak Molarity [nmol/l]	% Integrated Area	Peak Comment	Observations
15	7.28	-	747	-		Lower Marker
103	6.12	-	91.5	9.21		
180	5.51	-	47.1	8.28		
283	5.03	-	27.3	7.57		
380	4.66	-	18.8	7.00		
486	4.85	-	15.3	7.29		
576	8.62	-	23.1	12.97		
711	5.31	-	11.5	7.98		
811	4.43	-	8.40	6.65		
906	6.97	-	11.8	10.47		
1164	3.74	-	4.94	5.62		
1450	6.03	-	6.40	9.07		
1910	5.25	-	4.23	7.90		
10000	3.25	3.25	0.500	-		Upper Marker
