# Supplementary material for: GelInsight: Open-source software for large-sample DNA fragmentation quality control in gel electrophoresis images
Source: PLoS One. 2026 Jan 7;21(1):e0340374. doi: 10.1371/journal.pone.0340374 (PMC12779122; doi:10.1371/journal.pone.0340374)
Supplement: S3 File — (PDF) [file pone.0340374.s003.pdf]

TapeStation sonicated DNA analysis

D1000 ScreenTape assay results

(A) D1000 DNA ScreenTape: sample Information

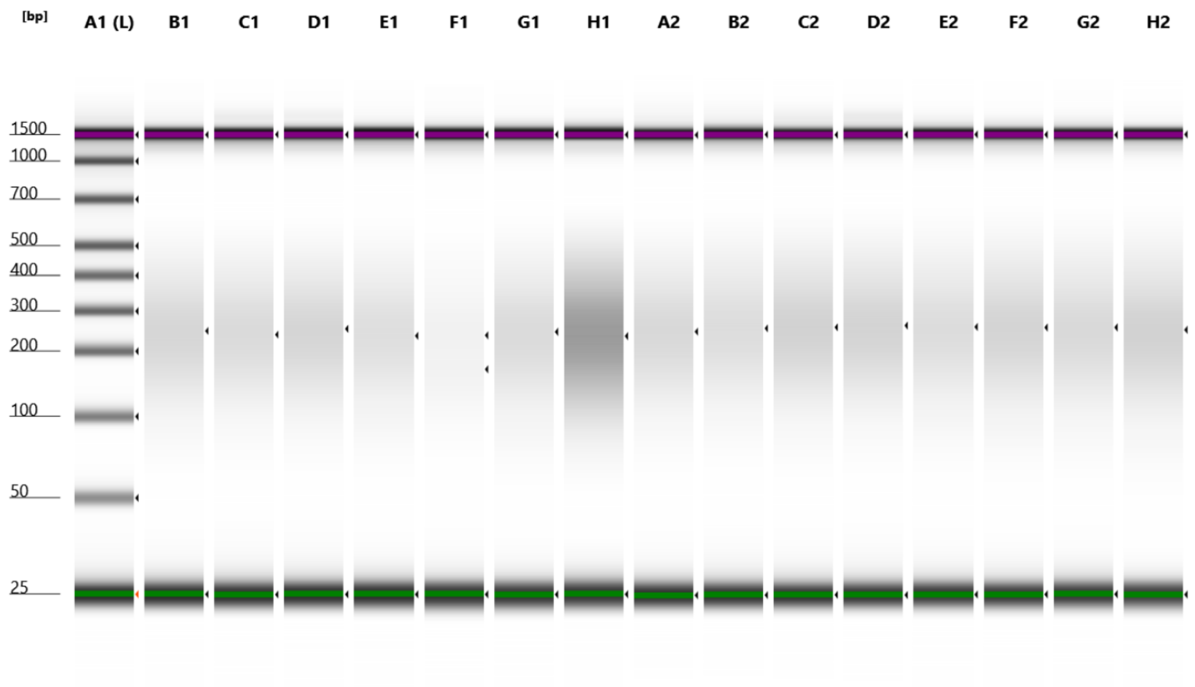

Default image (Contrast 100%)

Sample Info

Well	Conc. [ng/µl]	Sample Description
A1	14.4	Ladder
B1	1.49	1-1
C1	1.50	1-2
D1	1.43	1-3
E1	0.652	1-4
F1	0.458	1-5
G1	0.365	1-6
H1	4.89	1-7
A2	0.550	1-8
B2	1.56	2-1
C2	0.389	2-2
D2	0.297	2-3
E2	1.35	2-4
F2	0.484	2-5
G2	2.99	2-6
H2	2.39	2-7

(B) D1000 ScreenTape ladder

A1: Ladder

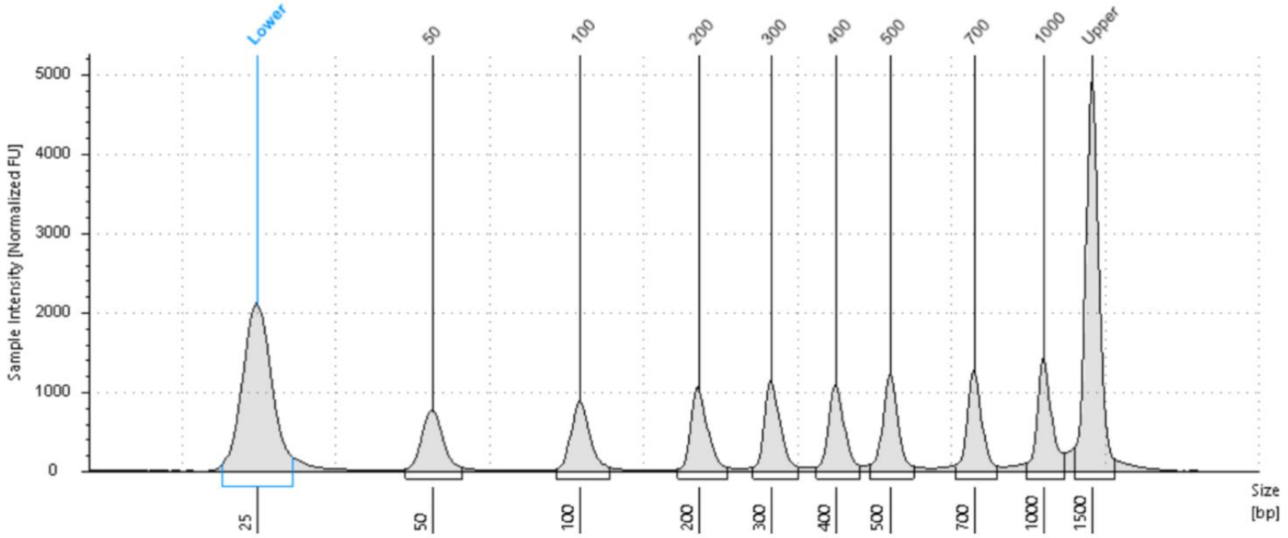

Size [bp]	Calibrated Conc. [ng/µl]	Assigned Conc. [ng/µl]	Peak Molarity [nmol/l]	% Integrated Area	Peak Comment	Observations
25	6.14	-	378	-		Lower Marker
50	1.64	-	50.4	11.36		
100	1.69	-	25.9	11.69		
200	1.80	-	13.8	12.46		
300	1.79	-	9.19	12.42		
400	1.76	-	6.77	12.21		
500	1.88	-	5.80	13.06		
700	1.86	-	4.08	12.86		
1000	2.01	-	3.09	13.94		
1500	6.50	6.50	6.67	-		Upper Marker

(C) sonicated DNA samples

B1: 1-1

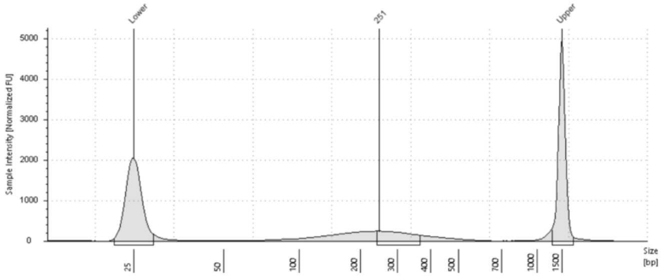

Size [bp]	Calibrated Conc. [ng/µl]	Assigned Conc. [ng/µl]	Peak Molarity [nmol/l]	% Integrated Area	Peak Comment	Observations
25	6.41	-	394	-		Lower Marker
251	1.49	-	9.16	100.00		
1500	6.50	6.50	6.67	-		Upper Marker

C1: 1-2

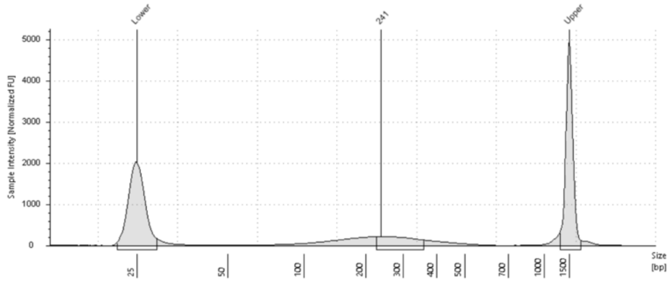

Size [bp]	Calibrated Conc. [ng/µl]	Assigned Conc. [ng/µl]	Peak Molarity [nmol/l]	% Integrated Area	Peak Comment	Observations
25	6.46	-	397	-		Lower Marker
241	1.50	-	9.57	100.00		
1500	6.50	6.50	6.67	-		Upper Marker

D1: 1-3

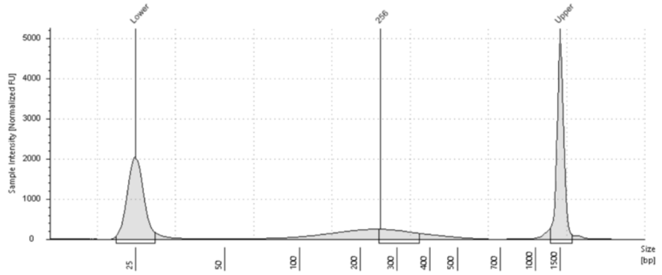

Size [bp]	Calibrated Conc. [ng/µl]	Assigned Conc. [ng/µl]	Peak Molarity [nmol/l]	% Integrated Area	Peak Comment	Observations
25	6.50	-	400	-		Lower Marker
256	1.43	-	8.59	100.00		
1500	6.50	6.50	6.67	-		Upper Marker

E1: 1-4

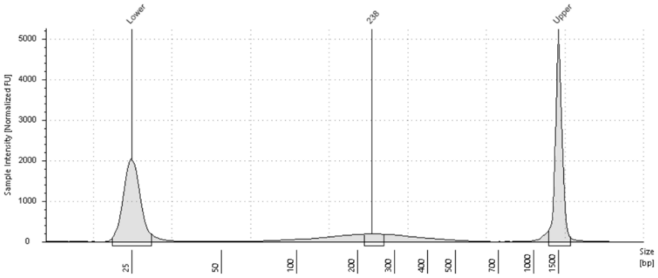

Size [bp]	Calibrated Conc. [ng/µl]	Assigned Conc. [ng/µl]	Peak Molarity [nmol/l]	% Integrated Area	Peak Comment	Observations
25	6.66	-	410	-		Lower Marker
238	0.652	-	4.21	100.00		
1500	6.50	6.50	6.67	-		Upper Marker

F1: 1-5

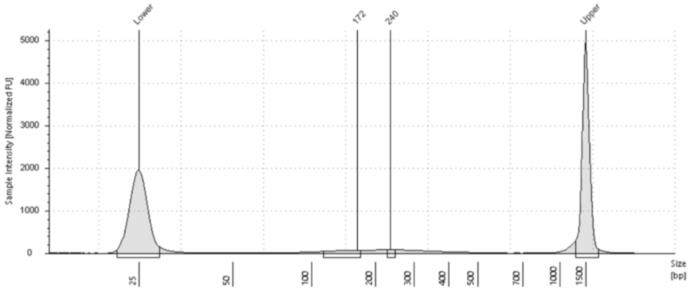

Size [bp]	Calibrated Conc. [ng/µl]	Assigned Conc. [ng/µl]	Peak Molarity [nmol/l]	% Integrated Area	Peak Comment	Observations
25	6.48	-	399	-		Lower Marker
172	0.360	-	3.21	78.43		
240	0.0989	-	0.635	21.57		
1500	6.50	6.50	6.67	-		Upper Marker

G1: 1-6

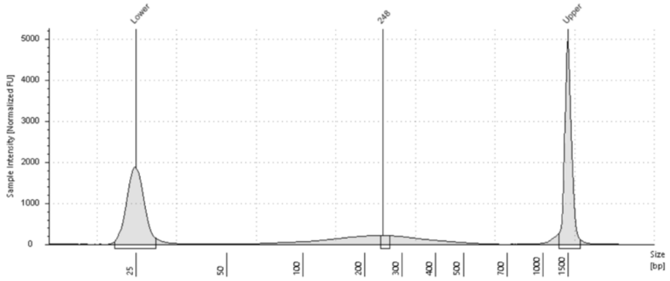

Size [bp]	Calibrated Conc. [ng/µl]	Assigned Conc. [ng/µl]	Peak Molarity [nmol/l]	% Integrated Area	Peak Comment	Observations
25	6.47	-	398	-		Lower Marker
248	0.365	-	2.26	100.00		
1500	6.50	6.50	6.67	-		Upper Marker

H1: 1-7

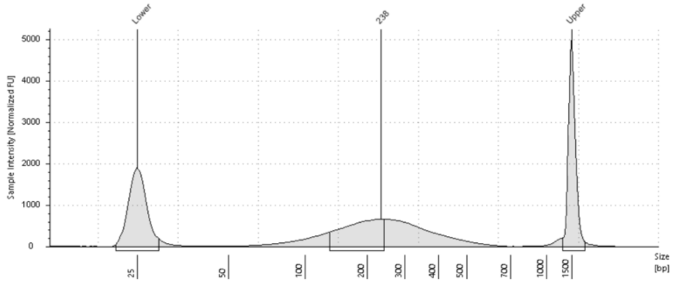

Size [bp]	Calibrated Conc. [ng/µl]	Assigned Conc. [ng/µl]	Peak Molarity [nmol/l]	% Integrated Area	Peak Comment	Observations
25	6.51	-	400	-		Lower Marker
238	4.89	-	31.6	100.00		
1500	6.50	6.50	6.67	-		Upper Marker

A2: 1-8

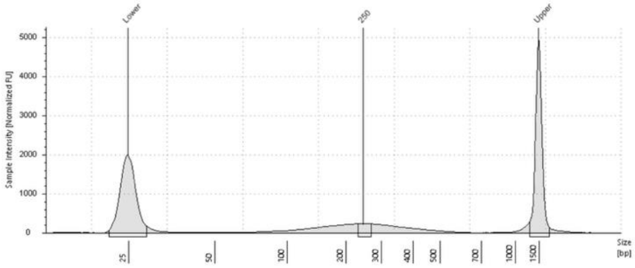

Size [bp]	Calibrated Conc. [ng/µl]	Assigned Conc. [ng/µl]	Peak Molarity [nmol/l]	% Integrated Area	Peak Comment	Observations
25	6.59	-	405	-		Lower Marker
250	0.550	-	3.38	100.00		
1500	6.50	6.50	6.67	-		Upper Marker

B2: 2-1

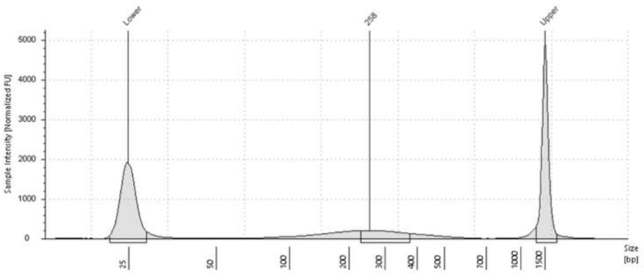

Size [bp]	Calibrated Conc. [ng/µl]	Assigned Conc. [ng/µl]	Peak Molarity [nmol/l]	% Integrated Area	Peak Comment	Observations
25	6.31	-	388	-		Lower Marker
258	1.56	-	9.29	100.00		
1500	6.50	6.50	6.67	-		Upper Marker

C2: 2-2

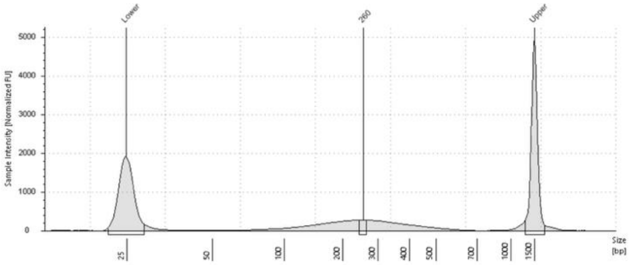

Size [bp]	Calibrated Conc. [ng/µl]	Assigned Conc. [ng/µl]	Peak Molarity [nmol/l]	% Integrated Area	Peak Comment	Observations
25	6.34	-	390	-		Lower Marker
260	0.389	-	2.30	100.00		
1500	6.50	6.50	6.67	-		Upper Marker

D2: 2-3

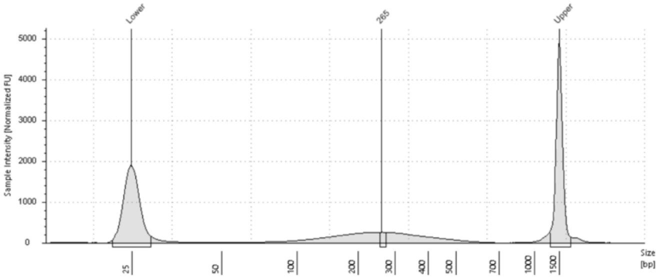

Size [bp]	Calibrated Conc. [ng/µl]	Assigned Conc. [ng/µl]	Peak Molarity [nmol/l]	% Integrated Area	Peak Comment	Observations
25	6.37	-	392	-		Lower Marker
265	0.297	-	1.73	100.00		
1500	6.50	6.50	6.67	-		Upper Marker

E2: 2-4

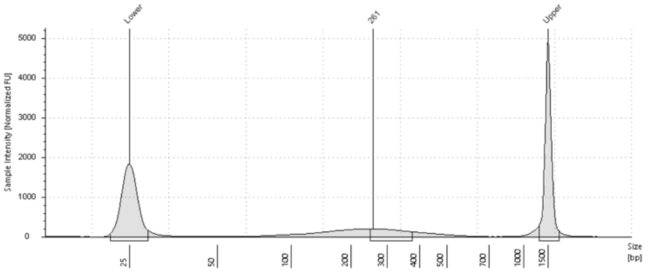

Size [bp]	Calibrated Conc. [ng/µl]	Assigned Conc. [ng/µl]	Peak Molarity [nmol/l]	% Integrated Area	Peak Comment	Observations
25	6.30	-	388	-		Lower Marker
261	1.35	-	7.96	100.00		
1500	6.50	6.50	6.67	-		Upper Marker

F2: 2-5

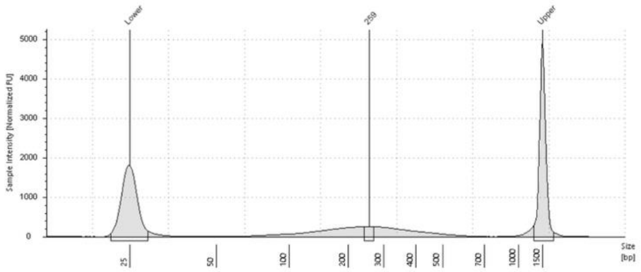

Size [bp]	Calibrated Conc. [ng/µl]	Assigned Conc. [ng/µl]	Peak Molarity [nmol/l]	% Integrated Area	Peak Comment	Observations
25	6.29	-	387	-		Lower Marker
259	0.484	-	2.87	100.00		
1500	6.50	6.50	6.67	-		Upper Marker

G2: 2-6

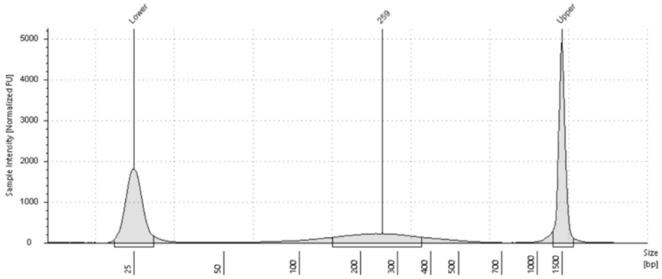

Size [bp]	Calibrated Conc. [ng/µl]	Assigned Conc. [ng/µl]	Peak Molarity [nmol/l]	% Integrated Area	Peak Comment	Observations
25	6.32	-	389	-		Lower Marker
259	2.99	-	17.8	100.00		
1500	6.50	6.50	6.67	-		Upper Marker

H2: 2-7

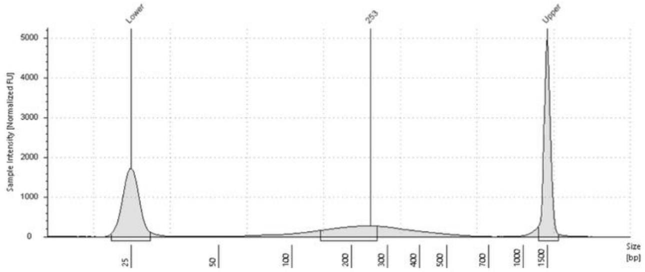

Size [bp]	Calibrated Conc. [ng/µl]	Assigned Conc. [ng/µl]	Peak Molarity [nmol/l]	% Integrated Area	Peak Comment	Observations
25	5.95	-	366	-		Lower Marker
253	2.39	-	14.5	100.00		
1500	6.50	6.50	6.67	-		Upper Marker

D1000 ScreenTape assay results

(D) D1000 DNA ScreenTape: sample Information

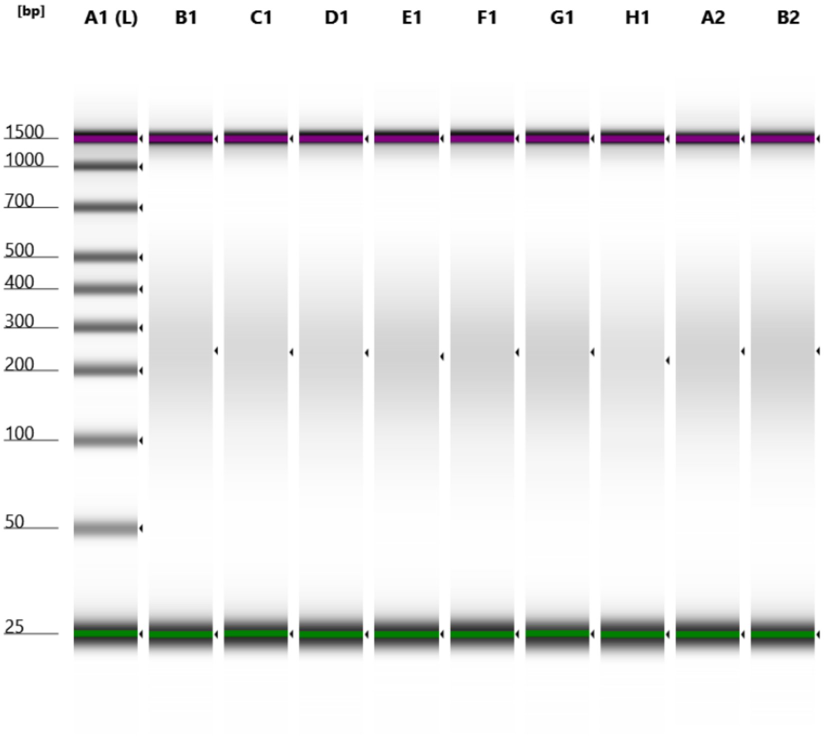

Default image (Contrast 100%)

Sample Info

Well	Conc. [ng/µl]	Sample Description
A1	14.4	Ladder
B1	1.47	2-8
C1	2.60	3-1
D1	0.242	3-2
E1	2.38	3-3
F1	0.294	3-4
G1	3.41	3-5
H1	0.186	3-6
A2	1.69	3-7
B2	0.489	3-8

(E) D1000 ScreenTape ladder

A1: Ladder

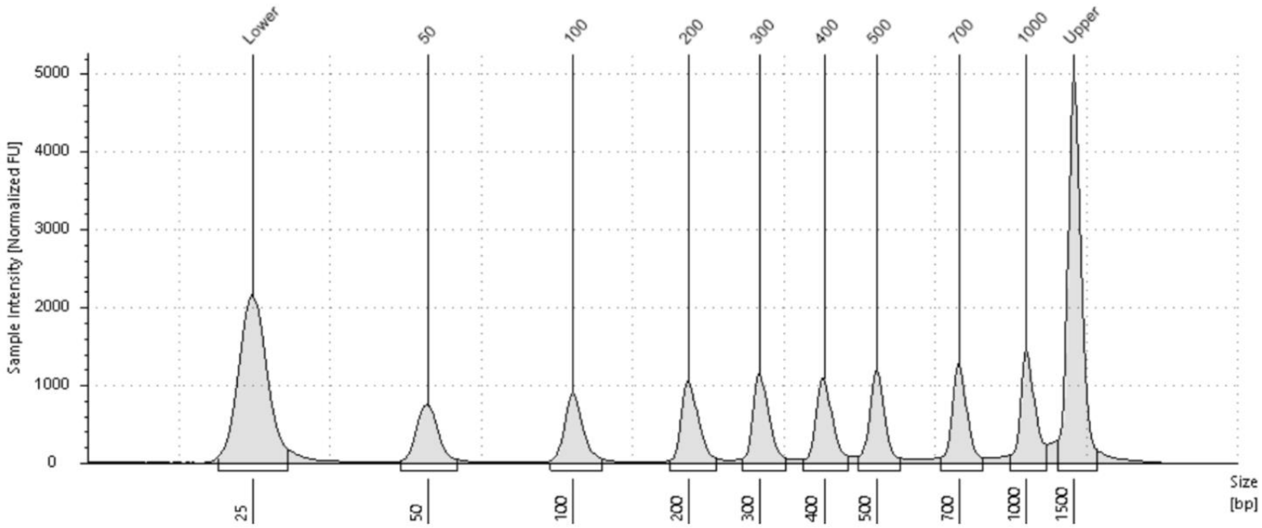

Size [bp]	Calibrated Conc. [ng/µl]	Assigned Conc. [ng/µl]	Peak Molarity [nmol/l]	% Integrated Area	Peak Comment	Observations
25	6.10	-	376	-		Lower Marker
50	1.62	-	49.8	11.23		
100	1.69	-	25.9	11.71		
200	1.80	-	13.8	12.48		
300	1.82	-	9.36	12.67		
400	1.79	-	6.87	12.40		
500	1.80	-	5.55	12.53		
700	1.86	-	4.09	12.93		
1000	2.03	-	3.12	14.06		
1500	6.50	6.50	6.67	-		Upper Marker

(F) sonicated DNA samples

B1: 2-8

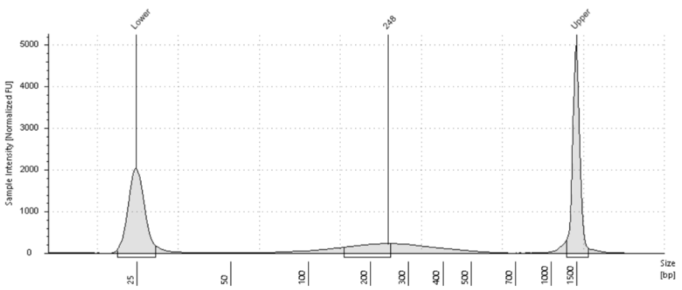

Size [bp]	Calibrated Conc. [ng/µl]	Assigned Conc. [ng/µl]	Peak Molarity [nmol/l]	% Integrated Area	Peak Comment	Observations
25	6.27	-	386	-		Lower Marker
248	1.47	-	9.16	100.00		
1500	6.50	6.50	6.67	-		Upper Marker

C1: 3-1

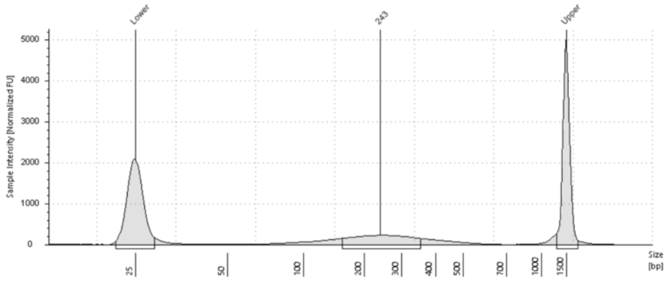

Size [bp]	Calibrated Conc. [ng/µl]	Assigned Conc. [ng/µl]	Peak Molarity [nmol/l]	% Integrated Area	Peak Comment	Observations
25	6.62	-	407	-		Lower Marker
243	2.60	-	16.4	100.00		
1500	6.50	6.50	6.67	-		Upper Marker

D1: 3-2

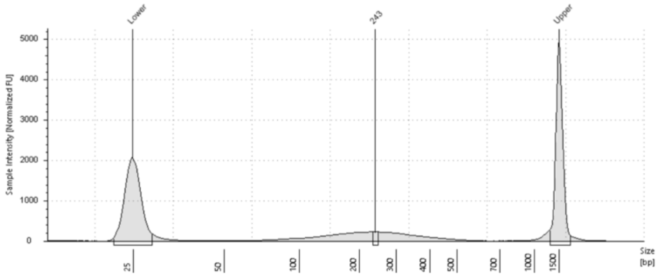

Size [bp]	Calibrated Conc. [ng/µl]	Assigned Conc. [ng/µl]	Peak Molarity [nmol/l]	% Integrated Area	Peak Comment	Observations
25	6.53	-	402	-		Lower Marker
243	0.242	-	1.53	100.00		
1500	6.50	6.50	6.67	-		Upper Marker

E1: 3-3

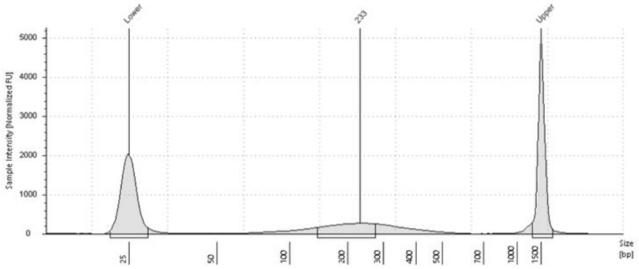

Size [bp]	Calibrated Conc. [ng/µl]	Assigned Conc. [ng/µl]	Peak Molarity [nmol/l]	% Integrated Area	Peak Comment	Observations
25	6.49	-	399	-		Lower Marker
233	2.38	-	15.7	100.00		
1500	6.50	6.50	6.67	-		Upper Marker

F1: 3-4

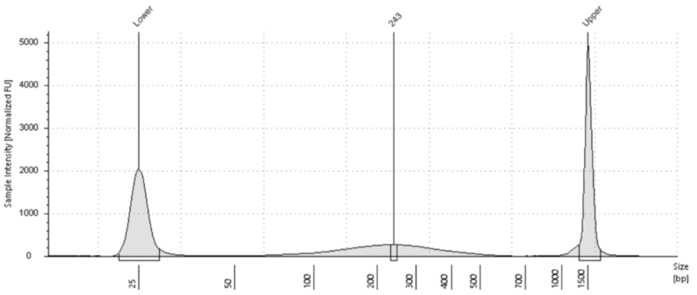

Size [bp]	Calibrated Conc. [ng/µl]	Assigned Conc. [ng/µl]	Peak Molarity [nmol/l]	% Integrated Area	Peak Comment	Observations
25	6.59	-	405	-		Lower Marker
243	0.294	-	1.86	100.00		
1500	6.50	6.50	6.67	-		Upper Marker

G1: 3-5

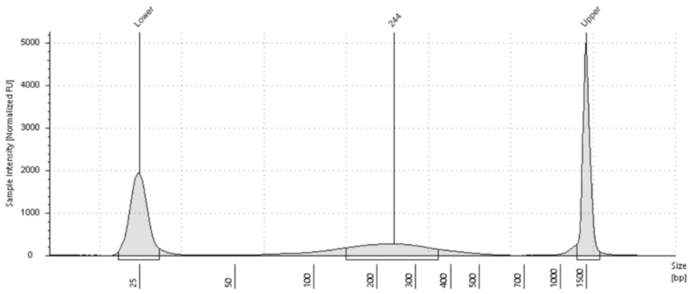

Size [bp]	Calibrated Conc. [ng/µl]	Assigned Conc. [ng/µl]	Peak Molarity [nmol/l]	% Integrated Area	Peak Comment	Observations
25	6.35	-	391	-		Lower Marker
244	3.41	-	21.5	100.00		
1500	6.50	6.50	6.67	-		Upper Marker

H1: 3-6

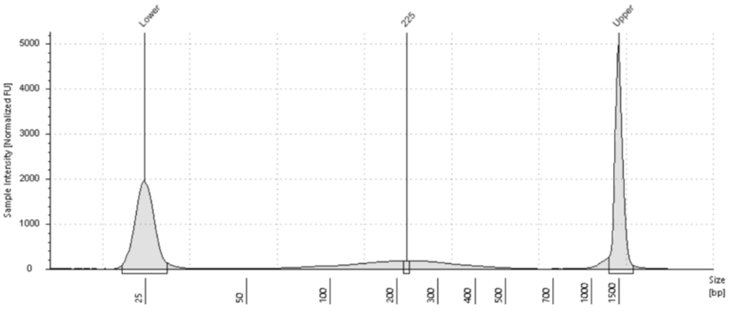

Size [bp]	Calibrated Conc. [ng/µl]	Assigned Conc. [ng/µl]	Peak Molarity [nmol/l]	% Integrated Area	Peak Comment	Observations
25	6.63	-	408	-		Lower Marker
225	0.186	-	1.27	100.00		
1500	6.50	6.50	6.67	-		Upper Marker

A2: 3-7

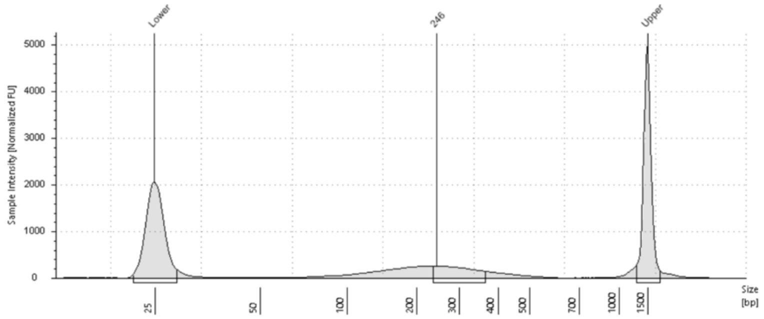

Size [bp]	Calibrated Conc. [ng/µl]	Assigned Conc. [ng/µl]	Peak Molarity [nmol/l]	% Integrated Area	Peak Comment	Observations
25	6.75	-	415	-		Lower Marker
246	1.69	-	10.6	100.00		
1500	6.50	6.50	6.67	-		Upper Marker

B2: 3-8

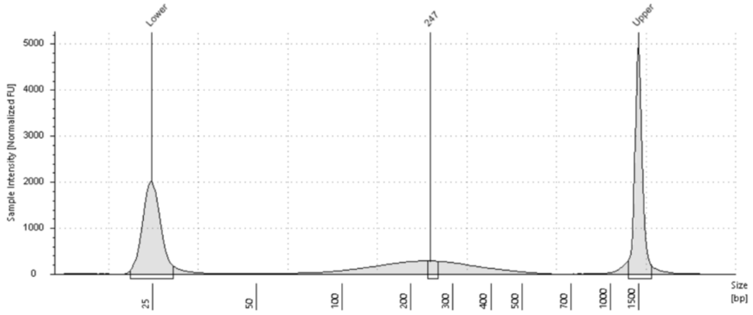

Size [bp]	Calibrated Conc. [ng/µl]	Assigned Conc. [ng/µl]	Peak Molarity [nmol/l]	% Integrated Area	Peak Comment	Observations
25	6.55	-	403	-		Lower Marker
247	0.489	-	3.05	100.00		
1500	6.50	6.50	6.67	-		Upper Marker

D5000 ScreenTape assay results

(G) D5000 DNA ScreenTape: sample Information

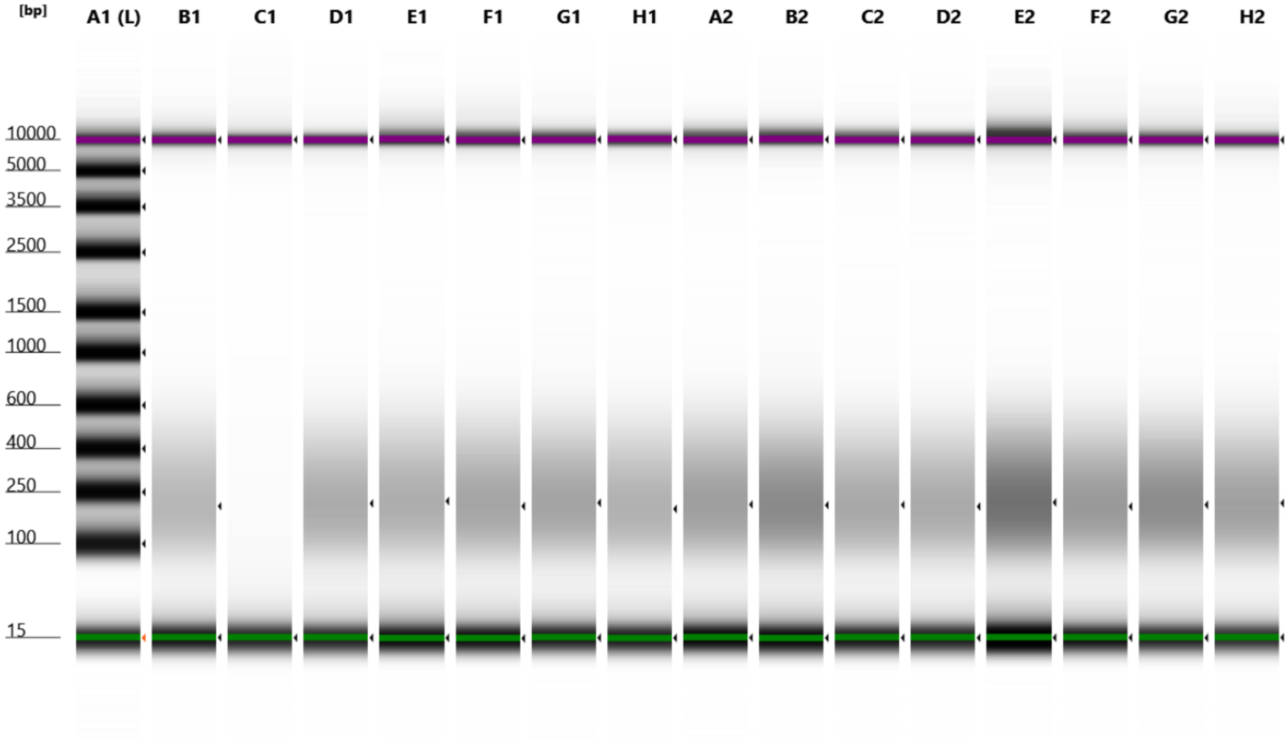

Default image (Contrast 100%)

Sample Info

Well	Conc. [ng/µl]	Sample Description
A1	94.6	Ladder
B1	1.05	1,1
C1		1,2
D1	8.09	1,3
E1	3.90	1,4
F1	3.58	1,5
G1	4.14	1,6
H1	3.23	1,7
A2	4.01	1,8
B2	9.33	2,1
C2	4.05	2,2
D2	3.63	2,3
E2	1.62	2,4
F2	4.19	2,5
G2	10.6	2,6
H2	5.21	2,7

(H) D5000 ScreenTape ladder

A1: Ladder

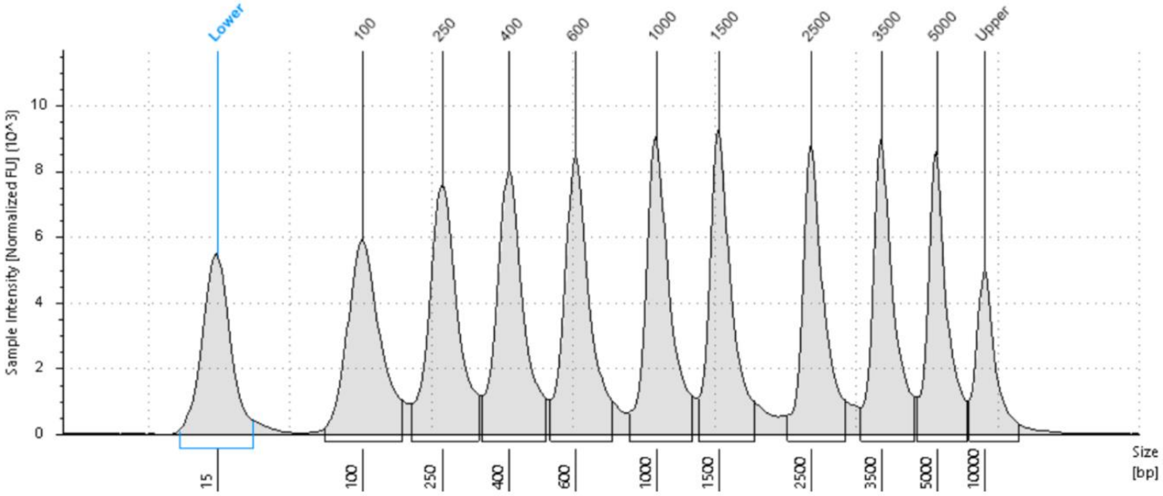

Size [bp]	Calibrated Conc. [ng/µl]	Assigned Conc. [ng/µl]	Peak Molarity [nmol/l]	% Integrated Area	Peak Comment	Observations
15	7.26	-	744	-		Lower Marker
100	10.3	-	159	10.90		
250	11.4	-	70.1	12.04		
400	11.3	-	43.5	11.96		
600	11.3	-	28.9	11.92		
1000	11.3	-	17.4	11.98		
1500	10.7	-	11.0	11.34		
2500	9.80	-	6.03	10.36		
3500	9.66	-	4.25	10.21		
5000	8.78	-	2.70	9.28		
10000	3.25	3.25	0.500	-		Upper Marker

(I) sonicated DNA samples

B1: 1,1

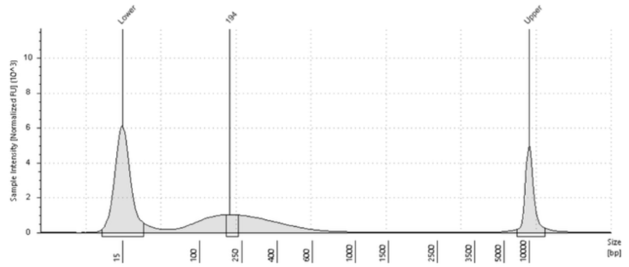

Size [bp]	Calibrated Conc. [ng/µl]	Assigned Conc. [ng/µl]	Peak Molarity [nmol/l]	% Integrated Area	Peak Comment	Observations
15	7.46	-	765	-		Lower Marker
194	1.05	-	8.35	100.00		
10000	3.25	3.25	0.500	-		Upper Marker

C1: 1,2

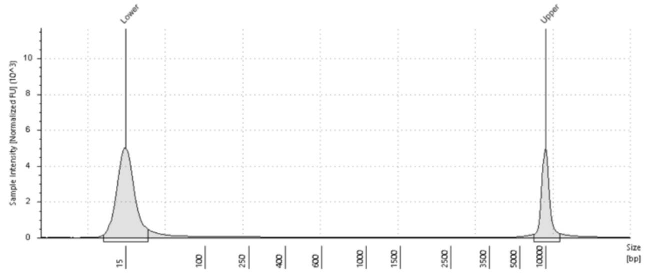

Size [bp]	Calibrated Conc. [ng/µl]	Assigned Conc. [ng/µl]	Peak Molarity [nmol/l]	% Integrated Area	Peak Comment	Observations
15	7.39	-	758	-		Lower Marker
10000	3.25	3.25	0.500	-		Upper Marker

D1: 1,3

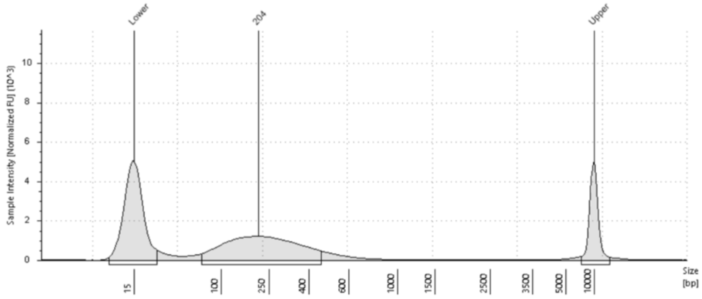

Size [bp]	Calibrated Conc. [ng/µl]	Assigned Conc. [ng/µl]	Peak Molarity [nmol/l]	% Integrated Area	Peak Comment	Observations
15	7.17	-	735	-		Lower Marker
204	8.09	-	60.9	100.00		
10000	3.25	3.25	0.500	-		Upper Marker

E1: 1,4

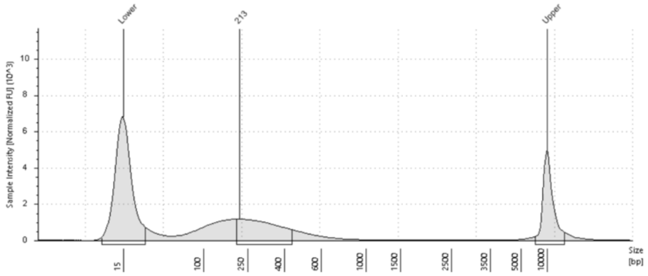

Size [bp]	Calibrated Conc. [ng/µl]	Assigned Conc. [ng/µl]	Peak Molarity [nmol/l]	% Integrated Area	Peak Comment	Observations
15	7.95	-	816	-		Lower Marker
213	3.90	-	28.1	100.00		
10000	3.25	3.25	0.500	-		Upper Marker

F1: 1,5

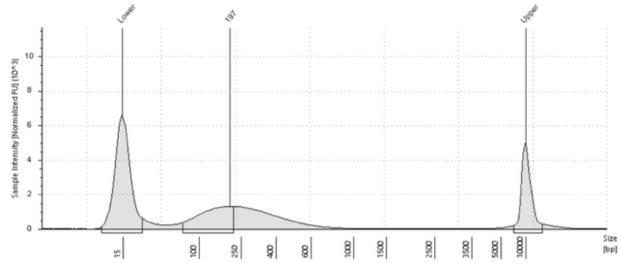

Size [bp]	Calibrated Conc. [ng/µl]	Assigned Conc. [ng/µl]	Peak Molarity [nmol/l]	% Integrated Area	Peak Comment	Observations
15	7.18	-	736	-		Lower Marker
197	3.58	-	28.0	100.00		
10000	3.25	3.25	0.500	-		Upper Marker

G1: 1,6

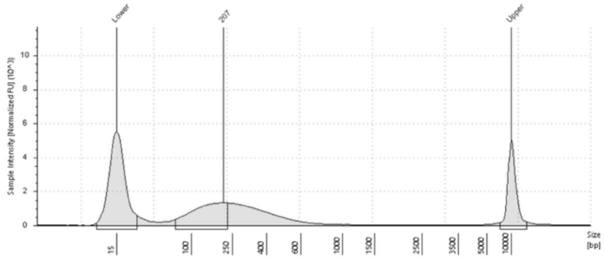

Size [bp]	Calibrated Conc. [ng/µl]	Assigned Conc. [ng/µl]	Peak Molarity [nmol/l]	% Integrated Area	Peak Comment	Observations
15	6.83	-	700	-		Lower Marker
207	4.14	-	30.8	100.00		
10000	3.25	3.25	0.500	-		Upper Marker

H1: 1,7

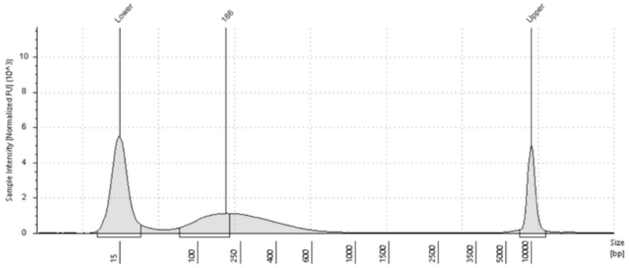

Size [bp]	Calibrated Conc. [ng/µl]	Assigned Conc. [ng/µl]	Peak Molarity [nmol/l]	% Integrated Area	Peak Comment	Observations
15	7.14	-	732	-		Lower Marker
186	3.23	-	26.8	100.00		
10000	3.25	3.25	0.500	-		Upper Marker

A2: 1,8

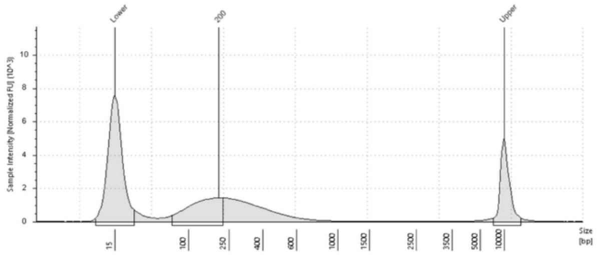

Size [bp]	Calibrated Conc. [ng/µl]	Assigned Conc. [ng/µl]	Peak Molarity [nmol/l]	% Integrated Area	Peak Comment	Observations
15	7.97	-	817	-		Lower Marker
200	4.01	-	30.8	100.00		
10000	3.25	3.25	0.500	-		Upper Marker

B2: 2,1

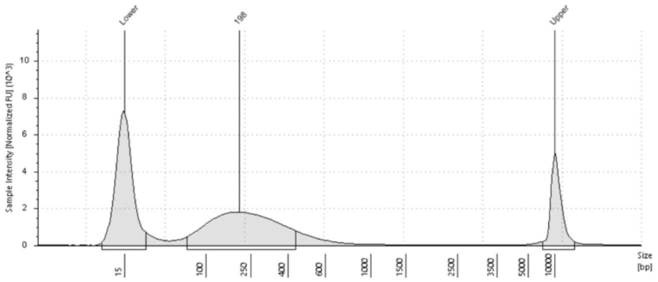

Size [bp]	Calibrated Conc. [ng/µl]	Assigned Conc. [ng/µl]	Peak Molarity [nmol/l]	% Integrated Area	Peak Comment	Observations
15	7.62	-	781	-		Lower Marker
198	9.33	-	72.3	100.00		
10000	3.25	3.25	0.500	-		Upper Marker

C2: 2,2

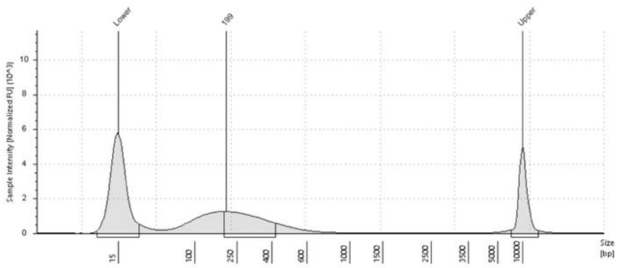

Size [bp]	Calibrated Conc. [ng/µl]	Assigned Conc. [ng/µl]	Peak Molarity [nmol/l]	% Integrated Area	Peak Comment	Observations
15	7.14	-	732	-		Lower Marker
199	4.05	-	31.3	100.00		
10000	3.25	3.25	0.500	-		Upper Marker

D2: 2,3

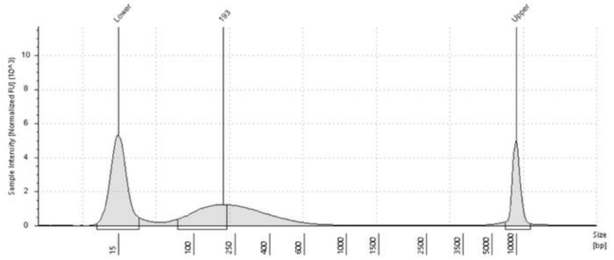

Size [bp]	Calibrated Conc. [ng/µl]	Assigned Conc. [ng/µl]	Peak Molarity [nmol/l]	% Integrated Area	Peak Comment	Observations
15	7.02	-	720	-		Lower Marker
193	3.63	-	28.8	100.00		
10000	3.25	3.25	0.500	-		Upper Marker

E2: 2,4

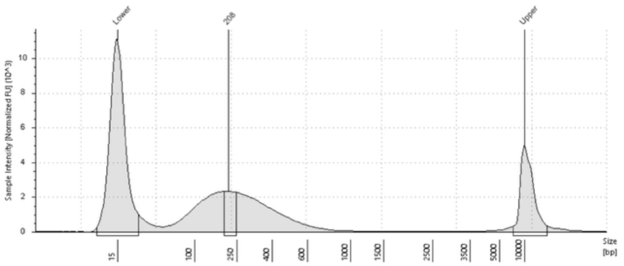

Size [bp]	Calibrated Conc. [ng/µl]	Assigned Conc. [ng/µl]	Peak Molarity [nmol/l]	% Integrated Area	Peak Comment	Observations
15	8.95	-	918	-		Lower Marker
208	1.62	-	12.0	100.00		
10000	3.25	3.25	0.500	-		Upper Marker

F2: 2,5

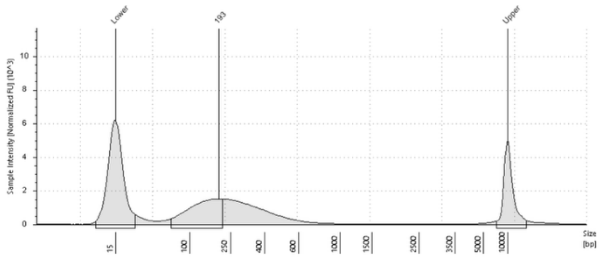

Size [bp]	Calibrated Conc. [ng/µl]	Assigned Conc. [ng/µl]	Peak Molarity [nmol/l]	% Integrated Area	Peak Comment	Observations
15	6.93	-	711	-		Lower Marker
193	4.19	-	33.3	100.00		
10000	3.25	3.25	0.500	-		Upper Marker

G2: 2,6

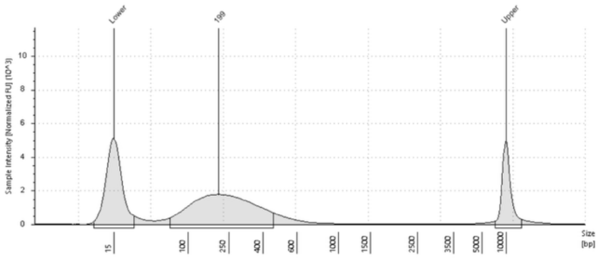

Size [bp]	Calibrated Conc. [ng/µl]	Assigned Conc. [ng/µl]	Peak Molarity [nmol/l]	% Integrated Area	Peak Comment	Observations
15	6.32	-	649	-		Lower Marker
199	10.6	-	82.0	100.00		
10000	3.25	3.25	0.500	-		Upper Marker

H2: 2,7

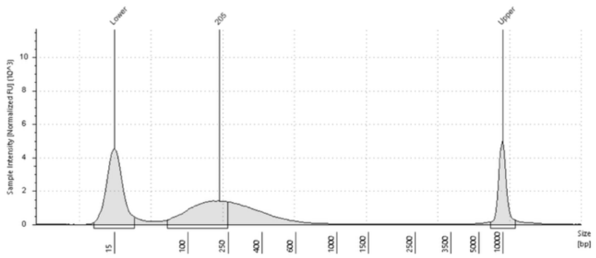

Size [bp]	Calibrated Conc. [ng/µl]	Assigned Conc. [ng/µl]	Peak Molarity [nmol/l]	% Integrated Area	Peak Comment	Observations
15	6.10	-	626	-		Lower Marker
205	5.21	-	39.1	100.00		
10000	3.25	3.25	0.500	-		Upper Marker

(J) D5000 DNA ScreenTape: sample Information

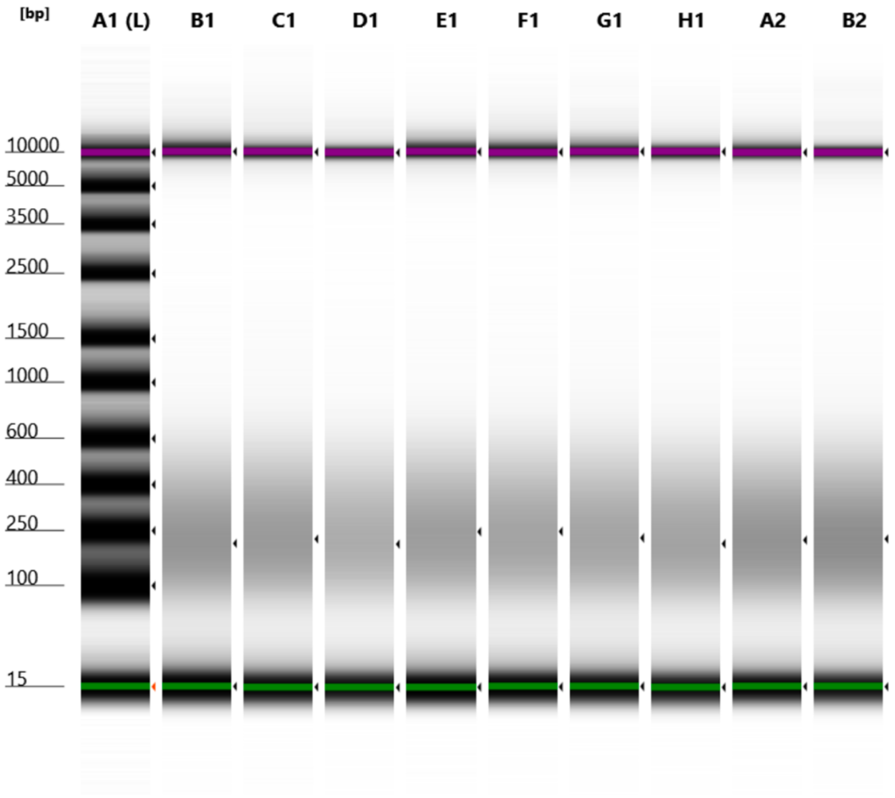

Default image (Contrast 100%)

Sample Info

Well	Conc. [ng/µl]	Sample Description
A1	80.1	Ladder
B1	0.748	2,8
C1	3.78	3.1
D1	2.99	3.2
E1	3.29	3,3
F1	3.07	3,4
G1	0.626	3,5
H1	6.72	3,6
A2	7.99	3,7
B2	1.05	3,8

(K) D5000 ScreenTape ladder

A1: Ladder

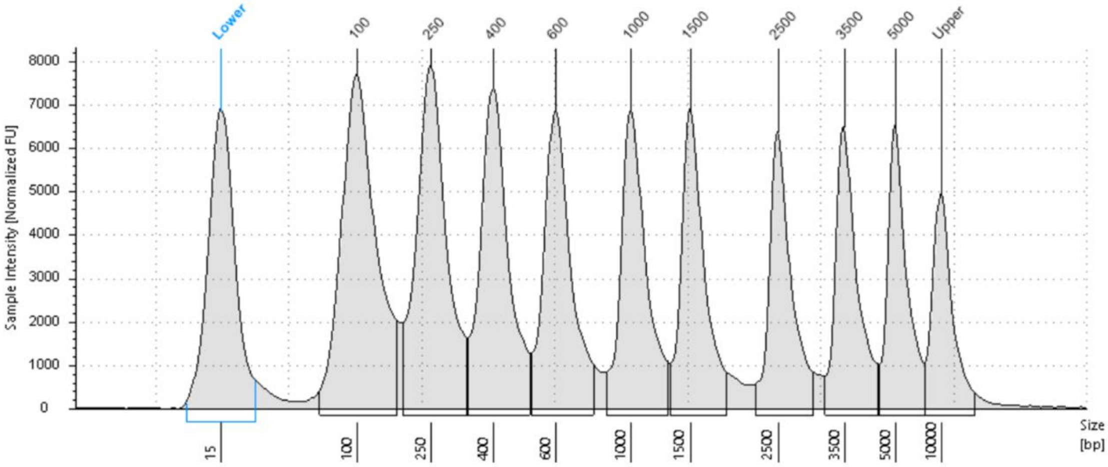

Size [bp]	Calibrated Conc. [ng/µl]	Assigned Conc. [ng/µl]	Peak Molarity [nmol/l]	% Integrated Area	Peak Comment	Observations
15	7.84	-	804	-		Lower Marker
100	13.1	-	202	16.36		
250	11.6	-	71.6	14.53		
400	10.1	-	38.9	12.62		
600	9.10	-	23.3	11.36		
1000	8.48	-	13.0	10.59		
1500	7.78	-	7.98	9.72		
2500	6.82	-	4.20	8.52		
3500	6.84	-	3.01	8.54		
5000	6.22	-	1.91	7.76		
10000	3.25	3.25	0.500	-		Upper Marker

(L) sonicated DNA samples

B1: 2,8

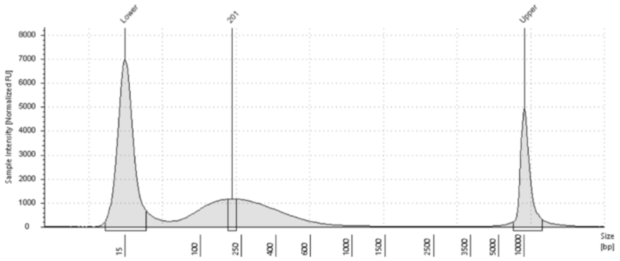

Size [bp]	Calibrated Conc. [ng/µl]	Assigned Conc. [ng/µl]	Peak Molarity [nmol/l]	% Integrated Area	Peak Comment	Observations
15	7.83	-	803	-		Lower Marker
201	0.748	-	5.74	100.00		
10000	3.25	3.25	0.500	-		Upper Marker

C1: 3.1

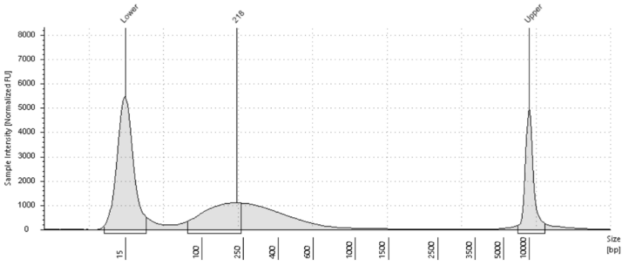

Size [bp]	Calibrated Conc. [ng/µl]	Assigned Conc. [ng/µl]	Peak Molarity [nmol/l]	% Integrated Area	Peak Comment	Observations
15	7.26	-	744	-		Lower Marker
218	3.78	-	26.7	100.00		
10000	3.25	3.25	0.500	-		Upper Marker

D1: 3.2

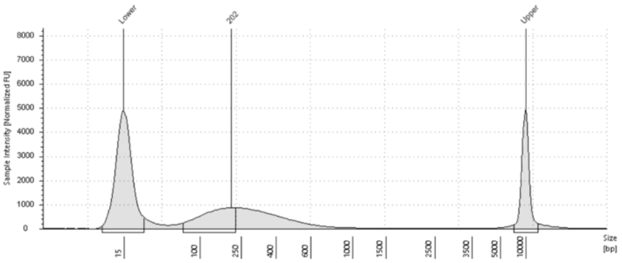

Size [bp]	Calibrated Conc. [ng/µl]	Assigned Conc. [ng/µl]	Peak Molarity [nmol/l]	% Integrated Area	Peak Comment	Observations
15	7.11	-	729	-		Lower Marker
202	2.99	-	22.8	100.00		
10000	3.25	3.25	0.500	-		Upper Marker

E1: 3,3

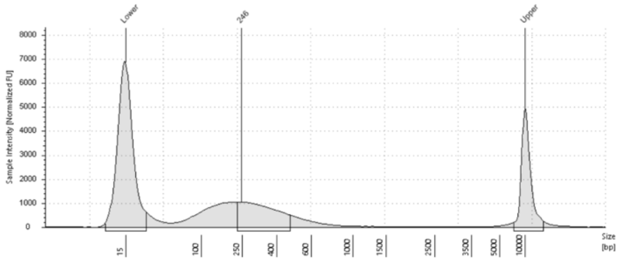

Size [bp]	Calibrated Conc. [ng/µl]	Assigned Conc. [ng/µl]	Peak Molarity [nmol/l]	% Integrated Area	Peak Comment	Observations
15	7.75	-	795	-		Lower Marker
246	3.29	-	20.6	100.00		
10000	3.25	3.25	0.500	-		Upper Marker

F1: 3,4

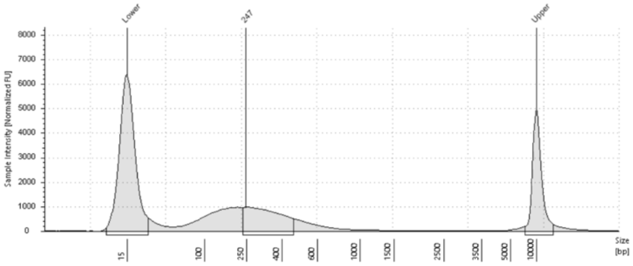

Size [bp]	Calibrated Conc. [ng/µl]	Assigned Conc. [ng/µl]	Peak Molarity [nmol/l]	% Integrated Area	Peak Comment	Observations
15	7.44	-	763	-		Lower Marker
247	3.07	-	19.1	100.00		
10000	3.25	3.25	0.500	-		Upper Marker

G1: 3,5

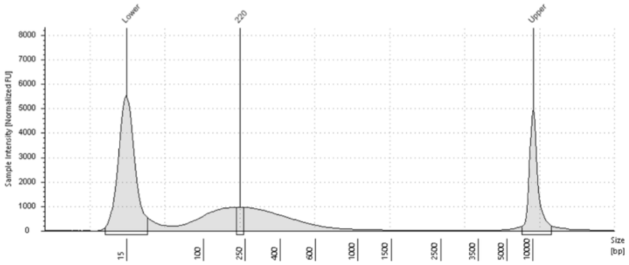

Size [bp]	Calibrated Conc. [ng/µl]	Assigned Conc. [ng/µl]	Peak Molarity [nmol/l]	% Integrated Area	Peak Comment	Observations
15	7.03	-	721	-		Lower Marker
220	0.626	-	4.38	100.00		
10000	3.25	3.25	0.500	-		Upper Marker

H1: 3,6

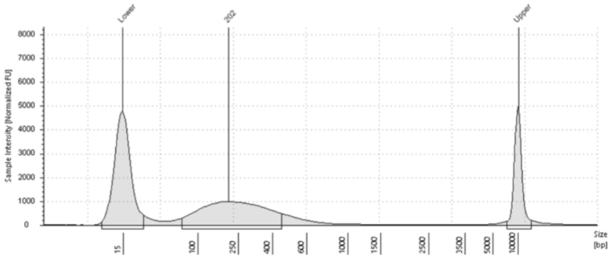

Size [bp]	Calibrated Conc. [ng/µl]	Assigned Conc. [ng/µl]	Peak Molarity [nmol/l]	% Integrated Area	Peak Comment	Observations
15	6.82	-	699	-		Lower Marker
202	6.72	-	51.2	100.00		
10000	3.25	3.25	0.500	-		Upper Marker

A2: 3,7

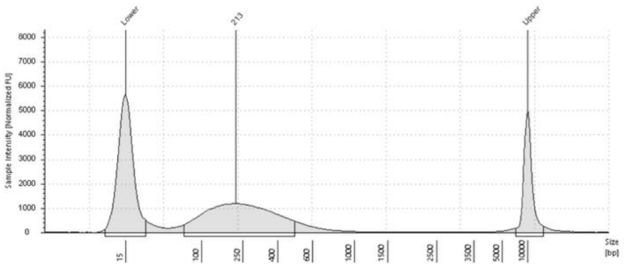

Size [bp]	Calibrated Conc. [ng/µl]	Assigned Conc. [ng/µl]	Peak Molarity [nmol/l]	% Integrated Area	Peak Comment	Observations
15	7.08	-	726	-		Lower Marker
213	7.99	-	57.6	100.00		
10000	3.25	3.25	0.500	-		Upper Marker

B2: 3,8

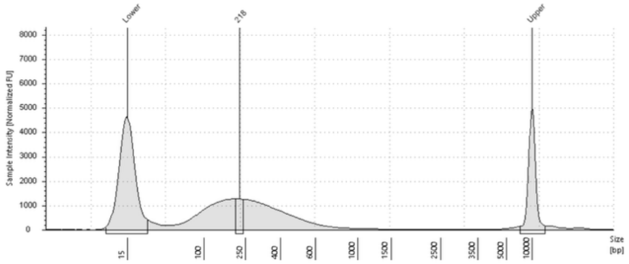

Size [bp]	Calibrated Conc. [ng/µl]	Assigned Conc. [ng/µl]	Peak Molarity [nmol/l]	% Integrated Area	Peak Comment	Observations
15	6.73	-	690	-		Lower Marker
218	1.05	-	7.41	100.00		
10000	3.25	3.25	0.500	-		Upper Marker
